# Diagnostic and Prognostic Significances of SOX9 in Thymic Epithelial Tumor

**DOI:** 10.3389/fonc.2021.708735

**Published:** 2021-10-28

**Authors:** Xiaodong Yuan, Lei Huang, Wenwu Luo, Yufei Zhao, Björn Nashan, Fazhi Yu, Yun Liu

**Affiliations:** ^1^ Organ Transplant Center, Department of Hepatobiliary and Transplantation Surgery, The First Affiliated Hospital of USTC, Division of Life Sciences and Medicine, University of Science and Technology of China, Hefei, China; ^2^ Department of Oncology, Ruijin Hospital, Shanghai Jiao Tong University School of Medicine, Shanghai, China; ^3^ Department of Pathology, Anhui Medical University, Hefei, China; ^4^ Department of Radiation Oncology, Anhui Provincial Cancer Hospital, The First Affiliated Hospital of USTC, Division of Life Sciences and Medicine, University of Science and Technology of China, Hefei, China; ^5^ The First Affiliated Hospital of USTC, School of Life Sciences, University of Science and Technology of China, Hefei, China

**Keywords:** SOX9, POU2F3, tuft cell, thymic epithelial tumor, tumor microenvironment

## Abstract

**Background:**

Thymic epithelial tumors (TETs) are rare tumors originating from the thymic epithelial cells. *SOX9*, a member of the family of *SOX* (SRY-related high-mobility group box) genes, has been considered as an oncogene and therapeutic target in various cancers. However, its role in TETs remains uncertain.

**Methods:**

Using the immunohistochemistry method, the expression of SOX9 was analyzed in TETs tissues, including 34 thymoma (8 cases with type A, 6 with type AB, 6 with type B1, 9 with type B2, and 5 with type B3 thymomas) and 20 thymic cancer tissues and the clinicopathologic and prognostic significances were evaluated. Further bioinformatics analysis of gene expression profiles of thymomas with high and low SOX9 expressions and the corresponding survival analyses were based on the thymoma cases identified in The Cancer Genome Atlas (TCGA) database, with the median expression level of SOX9 selected as cutoff.

**Results:**

Immunohistochemistry staining showed that SOX9 was highly expressed in the nuclei of the epithelial cells of the Hassall’s corpuscles and of the TET tumor cells. SOX9 expression was significantly associated with histological type and high expression indicated unfavorable clinical outcomes of thymomas. Bioinformatics analysis revealed that genes positively associated with SOX9 expression were mapped in proteoglycans in cancer, cell adhesion molecules, and molecules involved in extracellular matrix-receptor interaction and the TGF-β signaling pathway, and that genes negatively associated with SOX9 expression were mapped in molecules involved in primary immunodeficiency, the T cell receptor signaling pathway, Th17 cell differentiation, PD-L1 expression, and the PD-1 checkpoint pathway in cancer. In addition, SOX9 expression was positively associated with POU2F3 and TRPM5 expressions, the master regulators of tuft cells, suggesting that high SOX9 expression might be associated with the tuft cell phenotype of thymomas. Moreover, high SOX9 expression was associated with immune dysregulation of thymoma, and M2 macrophage significantly dominated in the high SOX9 expression group.

**Conclusion:**

SOX9 may serve as a diagnostic and prognostic marker for TETs. Notably, high SOX9 expression in TETs may indicate a tuft cell phenotype and an immune suppressive microenvironment of thymomas.

## Introduction

Thymic epithelial tumors (TETs), including thymomas and thymic carcinomas, are rare tumors of the mediastinum and originate from the thymic epithelial cells (1, 2). Histologically, most of thymic tumors are composed of non-malignant-appearing thymic epithelial cells mixed with multiple proportions of lymphocytes, which makes it difficult to diagnose and predict the prognosis of thymic tumors (1). According to the World Health Organization (WHO) classification, thymic epithelial cells are categorized into six subtypes, including A, AB, B1, B2, B3, and C (also known as TCs) based on histological appearance (3–5). Nevertheless, TETs are regarded as malignant tumors regardless of subtype or histology. According to histological subtype, types A, AB, and B1 have an excellent overall survival rate of 90–95% at 10 years; The 5-year survival rates for types B2, B3, and C are 75%, 70%, and 48%, respectively (1). Surgery remains the main treatment followed by adjuvant radiation therapy for diseases invading surrounding tissues (6, 7). For patients with inoperable refractory or recurrent diseases, postoperative systemic chemotherapy is currently recommended (2). However, there is still a lack of standard treatment strategy after first-line failure. Hence, the exact pathologic diagnosis of TETs is essential for determining the treatment strategy and predicting the prognosis. Although some biomarkers have been identified for the diagnosis of TETs, more valuable markers for the diagnosis and prognosis prediction of TETs are urgently needed.

*SOX9*, a member of the family of *SOX* (SRY-related high-mobility group box) genes, exerts regulatory functions in multiple organs development, cell-fate decision, and differentiation (8). Accumulating studies have demonstrated that SOX9 is also involved in the development of multiple cancers, including gastrointestinal, breast, brain, urological, and lung cancers and others (9–13). Collectively, SOX9 plays critical roles in tumor development and progression, including tumor initiation, tumor microenvironment regulation, metastasis, and drug resistance (14, 15).

Recently, using the immunohistochemistry method, we found that SOX9 was highly expressed in the epithelial component of thymus, especially in the epithelial cells of Hassall’s corpuscles. Moreover, SOX9 was observed to be highly expressed in the nuclei of TET tumor cells and may serve as a diagnostic marker for thymomas. However, the molecular function of SOX9 in TETs has not been well documented yet. In this study, we first examined the expression of SOX9 in TETs to evaluate whether SOX9 could serve as a diagnostic marker for TETs. In addition, using bioinformatics methods, we further investigated the potential function of SOX9 in the development of TETs.

## Materials and Methods

### Human Specimens and Ethics Approval

This study enrolled 34 thymoma (including 8 cases with type A, 6 with type AB, 6 with type B1 9 with type B2, and 5 with type B3 thymomas) and 20 thymic carcinoma tissues from the First Affiliated Hospital of Anhui Medical University (Hefei, China). This study was approved by the local ethics committees.

### Immunohistochemistry and Staining Evaluation

Immunohistochemical staining was performed as previously described (16). Briefly, the sections were deparaffinized in serial ethanol dilutions and rehydrated. Heat-induced antigen retrieval was performed with 0.01 M sodium citrate buffer (pH=6.0) at 98°C for 10 min. Endogenous peroxidase activity was blocked with 3% hydrogen peroxide in distilled water for 10 min, followed by pre-incubation in 5% normal goat serum to block nonspecific staining for 30 min at room temperature to prevent unspecific binding of antibodies. The tissue sections were incubated with polyclonal rabbit anti-SOX9 antibody (AB5535; Sigma-Aldrich) at a dilution of 1:100 for 4 h at room temperature. The specimens were subsequently washed in phosphate buffered saline and incubated with anti-rabbit secondary antibody conjugated with horse radish peroxidase for 1 h at room temperature, and then detected with 3, 3’-diaminobenzidine for 8 min. After being counterstained with hematoxylin, all sections were dehydrated and mounted with malinol mounting medium. Immunostaining results for SOX9 were scored semi-quantitatively based on the intensity and proportion of positive tumor cell nuclei as previously described (16). In detail, the intensity score of nuclear SOX9 staining was classified into four grades: 0, negative; 1, weak with yellow color; 2, medium with brown color; 3, strong with black color. The proportion score of SOX9 positive cell nuclei was evaluated under 200X magnification and was defined as 4 grades: 0, no positive cells; 1, positive cells: ≤ 30%; 2, 30% < positive cells ≤ 60%; 3, 60% < positive cells. The final immunostaining scores were evaluated by multiplying the intensity score and proportion score. The samples with final scores over 3 were identified as high SOX9 expression, and the others were identified as low SOX9 expression.

### Database

Gene expression data and clinical features of TET samples (including 108 thymomas and 11 thymic carcinomas) were collected from the publicly available datasets of The Cancer Genome Atlas (TCGA) (https://tcga-data.nci.nih.gov/tcga/).

### Identification of Differentially Expressed Genes (DEGs)

The R software (https://www.r-project.org/) and the *limma* package were utilized to identify DEGs by comparing cases with high and low SOX9 expressions. Gene expression with |log_2_(fold-change) | > 2 and an adjusted *P* < 0.05 was considered as significant.

### Gene Set Enrichment Analysis Heat-Maps, Volcano Plots, and Venn Diagrams

Heat-maps of the top 100 DEGs were constructed using the GSEA software (version 4.0.3) by the Broad Institute [Morpheus (broadinstitute.org)] (17) and the volcano plots of DEGs were generated using the GraphPad Prism software (version 5.03; GraphPad Software Inc.). To identify genes in DEGs regulated by transcription factor SOX9, the potential target genes of SOX9 were download from the ChIP Enrichment Analysis (CHEA) databases (https://maayanlab.cloud/Harmonizome/dataset/CHEA+Transcription+Factor+Targets) which was designed for the identification of target genes of transcription factors from published ChIP-chip, ChIP-seq, and other transcription factor binding site profiling studies (18). Then, the overlap of DEGs and potential target genes of SOX9 identified from the CHEA dataset were performed by Venn diagrams which was created using an online analysis tool (https://bioinfogp.cnb.csic.es/tools/venny/index.html) (18, 19).

### Enrichment Analysis of Gene Ontology and Kyoto Encyclopedia of Genes and Genomes (KEGG) OF DEGs

The *clusterProfiler*, *org.Hs.eg.db*, *enrichplot*, and *ggplot2* packages in the R software were used to perform the GO and KEGG enrichment analyses of DEGs. GO categories, including biological processes (BPs), molecular functions (MFs), and cellular components (CCs), were analyzed. *P*- and q-values <0.05 were regarded to indicate significant enrichment.

### Protein-Protein Interaction (PPI) Network Construction and Prediction of SOX9 Binding Sites Within the POU2F3 Promoter

PPI network was constructed by STRING database (http://string-db.org/) (20). Nodes with confidence of interactive relationship larger than 0.40 were used for building network. Disconnected nodes were hidden in the network. The JASPAR (http://jaspar.genereg.net) was used to predict the potential binding sites of *SOX9* within the promoter of POU2F3 by using the deposited *SOX9* binding site matrix profile MA0077.1 (21). According to the Ensembl deposited gene sequence, nucleotides have been analyzed from the 2000 upstream of the transcription starting site of POU2F3.

### Tumor Immune Estimation Resource Database Analysis (TIMER) and Tumor-infiltrating Immune Cells Profile

TIMER is a comprehensive website for the automatic analysis and visualization of the associations between immune infiltration levels and a series of variables (https://cistrome.shinyapps.io/timer/) (22, 23). The cell-type identification by estimating relative subsets of RNA transcripts (CIBERSORT) is a computational method which is applied for estimating the TIC abundance profile in all thymoma tumor samples (24). Tumor immune and stromal scores as well as microenvironment scores were used to predict the level of infiltrating stromal and immune cells as well as tumor purity and evaluated by CIBERSORT online software (http://cibersort.stanford.edu/). The abundances of six types of immune cells (CD4+ T cells, CD8+ T cells, B cells, neutrophils, dendritic cells, and macrophages) were evaluated by the TIMER algorithm and the abundances of M1 and M2 macrophages were evaluated by the CIBERSORT algorithm based on gene expression data of thymomas from TCGA database.

### Statistics Analyses

Continuous results were expressed as mean ± standard deviation (SD). Two-tailed unpaired Student *t*-test was used to compare continuous variables between groups. The chi-square test or Fisher’s exact probability method were used to evaluate the correlation between SOX9 expression and clinical characteristics of patients. The associations of SOX9 expression with the expression of the indicated genes and the tumor microenvironment, stromal, and immuno-scores were analyzed using Parametric Correlation and the Pearson correlation (r) was calculated to evaluate the fitting strength for each correlation. All data analyses were conducted using the GraphPad Prism software (version 5.03; GraphPad Software Inc.) if not otherwise specified. Findings with *P* values less than 0.05 were considered significant.

The 108 thymoma cases from the TCGA thymoma database were categorized into high and low SOX9 expression groups using the median SOX9 expression level as threshold for survival analysis, where the Kaplan-Meier method was used and survival between groups were compared using the log-rank test with two-sided *P* < 0.05 indicating statistical significance.

## Results

### Immunohistochemical Staining of SOX9 Expression in Thymic Tumors

**Figure 1:**
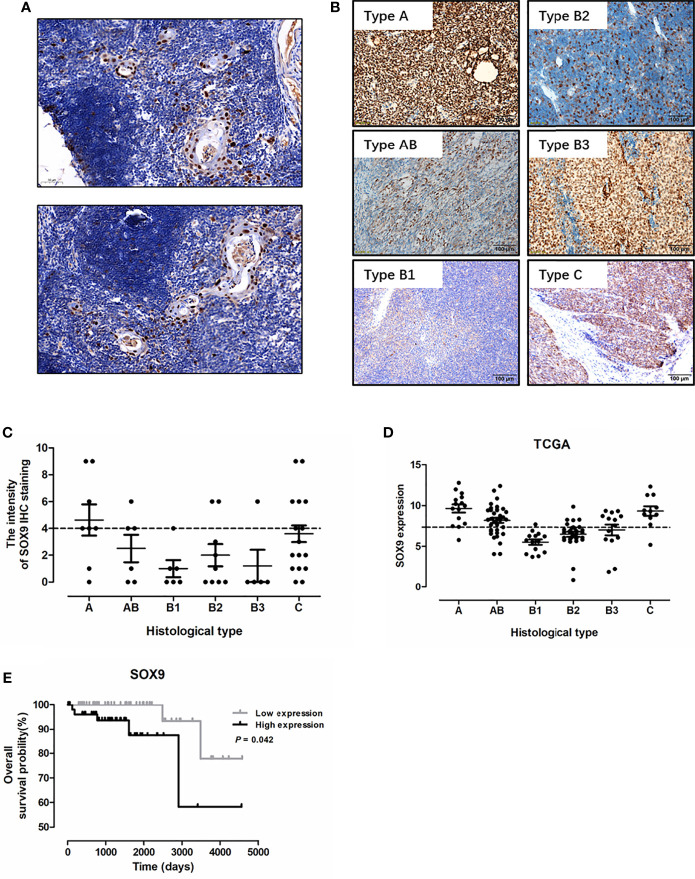
SOX9 expression in thymic epithelial tumors and its prognostic significance. **(A)** Representative immunostaining images for SOX9 expression in the epithelial cells of Hassall’s corpuscles in the non-tumor area of thymic epithelial tumors. **(B)** Representative immunostaining images for SOX9 in different histological types of thymomas and thymic carcinomas. **(C)** The distribution of SOX9 expression intensity quantified by IHC staining in thymomas and thymic carcinomas in our cohort. **(D)** The distribution of SOX9 RNA expression in thymomas and thymic carcinomas in the TCGA dataset. **(E)** Kaplan–Meier survival curves showing that thymoma patients from TCGA dataset with high SOX9 RNA expression had shorter overall survival time (*P* < 0.05), as determined by the log-rank test. Thymoma patients (n = 108) were categorized into high and low SOX9 expression groups with the median value of SOX9 mRNA expression level as cutoff.

To investigate the diagnostic significance of SOX9 in thymic tumors, immunohistochemistry staining of SOX9 expression was performed in 34 thymomas (including 8 cases with type A, 6 with type AB, 6 with type B1, 9 with type B2, and 5 with type B3 thymomas) and 20 thymic carcinoma tissues. The representative staining patterns of SOX9 in the non-tumor area of TETs are shown in **Figure 1A**; SOX9 expression patterns in TET tumor tissues are shown in **Figure 1B**. SOX9 was observed to be expressed in the nuclei of thymic epithelial cells and tumor cells. SOX9 staining intensity in TET tissues is shown in **Figure 1C**. The ratios of strong SOX9 staining in different types of thymomas and thymic carcinomas are summarized in **Table 1**. Specifically, strong staining of SOX9 was observed in 6 of 8 (70%) cases with type A, 3 of 6 (50.00) of cases with type AB, 2 of 9 (22.22%) cases with type B2 thymomas, and 9 of 20 (45%) cases with thymic carcinomas. The ratios of strong SOX9 staining in the other types were all smaller than 33%.

**Table 1:** Correlation between SOX9 protein expression level and clinicopathological parameters of patients with thymomas in our cohort.

Clinicopathological parameters	SOX9 staining		*P*
	low (n = 21)	high (n = 13)	
Age (years, mean ± SD)	52.29 ± 11.67	60.69 ± 13.87	0.0749
Sex			0.2793
Male	11 (52.38%)	10 (47.62%)	
Female	10 (76.92%)	3 (23.07%)	
Histological type			0.0131
A	2 (25.00%)	6 (75.00%)	
AB	3 (50.00%)	3 (50.00%)	
B1	5 (83.33%)	1 (16.67%)	
B2	7 (77.78%)	2 (22.22%)	
B3	4 (80.00%)	1 (20.00%)	

SD, standard deviation.

### SOX9 Expression Was Associated With Histological Types of Thymoma

We analyzed the association of SOX9 expression with clinical parameters of thymoma patients, including age, sex, and histological type (**Table 1**), and found that SOX9 expression tended to correlate with the histological type of thymomas (**Table 1**, *P* = 0.0131). To further verify the clinical significance of SOX9 expression in TETs, we examined SOX9 expression using the RNA-seq data of thymomas in TCGA. **Figure 1D** shows the SOX9 mRNA expressions in different types of thymomas (n = 108) and thymic carcinomas (n = 11).

Then, SOX9 expression in thymomas was categorized into high and low expression groups, with the median level of SOX9 expression selected as the cutoff. High SOX9 expression was observed in 14 of 15 (93%) cases with type A, 24 of 36 (67%) cases with type AB, and 8 of 14 (57%) cases with type B3 thymomas. The ratios of high SOX9 expression in all the other types were smaller than 30%. In addition, SOX9 was also highly expressed in thymic carcinoma (**Figures 1B–D**). Then, we evaluated the association of SOX9 expression with the clinical and pathologic parameters of thymoma cases from TCGA dataset, including age, sex, histological type, history of myasthenia gravis, Masaoka stage, radiation therapy, and new tumor event after initial treatment, and found that SOX9 expression was correlated with the histological type of thymomas (**Table 2**, *P* < 0.001). In addition, the ratio of patient received radiation therapy after operation was higher in patients with low SOX9 expression group (**Table 2**, *P* < 0.012). Moreover, survival analysis revealed that patients with high SOX9 expression had shorter median overall survival time (**Figure 1E**). These results suggested that SOX9 expression was associated with the histological type of thymomas and might serve as an unfavorable prognostic marker for thymomas.

**Table 2:** Correlation between SOX9 RNA expression and clinicopathological parameters of patients with thymomas in the TCGA cohort.

Clinicopathological parameters	SOX9		*P*
	Low (n = 54)	High (n = 54)	
Age (years, mean ± SD)	54.65 ± 12.78	59.89 ± 12.92	0.038
Sex			0.847
Male	29 (49.15%)	30 (50.85%)	
Female	25 (51.02%)	24 (48.98%)	
Histological type			<0.001
A	1 (6.67%)	14 (93.33%)	
AB	12 (33.33%)	24 (66.67%)	
B1	13 (92.86%)	1 (7.14%)	
B2	22 (75.86%)	7 (24.14%)	
B3	6 (42.86%)	8 (57.14%)	
History of myasthenia gravis			0.294
No	34 (47.89%)	37 (52.11%)	
Yes	20 (58.82%)	14 (41.18%)	
NA	0	3	
Masaoka stage			0.532
I-II	47 (52.22%)	43 (47.78%)	
III-IV	7 (43.75%)	9 (56.25%)	
NA	0	2	
Radiation therapy			0.012
No	29 (40.85%)	42 (59.15%)	
Yes	24 (66.67%)	12 (33.33%)	
NA	1	0	
New tumor event after initial treatment			0.221
No	46 (47.92%)	50 (52.08%)	
Yes	8 (66.67%)	4 (33.33%)	

TCGA, The Cancer Genome Atlas; SD, standard deviation; NA, not available.

### SOX9 Expression Was Associated With the Epithelial Cell Phenotype of Thymoma

**Figure 2:**
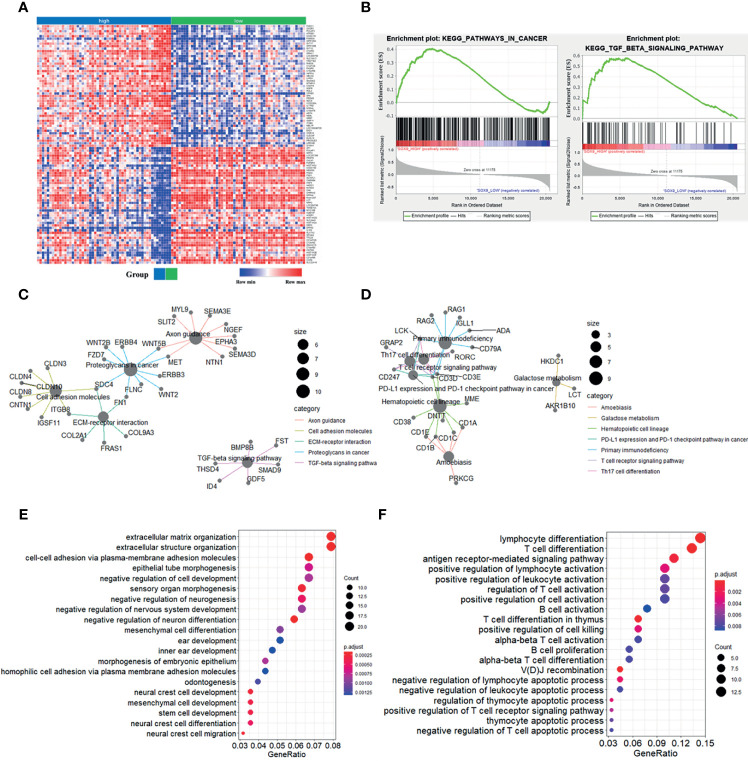
Enrichment analyses of genes significantly correlated with SOX9 expression in thymomas. **(A)** Heat-map of the top 100 upregulated and downregulated genes determined by the Gene Set Enrichment Analysis (GSEA) analysis. **(B)** GSEA analysis revealed enrichment of the gene sets related to the “pathway in cancer and TGF-β signaling pathway” in patients with high SOX9 expression. **(C)** The cnetplot depicted the five enriched Kyoto Encyclopedia of Genes and Genomes (KEGG) pathways detected and their associated differentially-expressed genes in thymoma patients with high SOX9 expression. **(D)** The cnetplot depicts the seven enriched KEGG pathways detected and their associated differentially-expressed genes in thymoma patients with low SOX9 expression. **(E, F)** The dot-plot depicts the activity of the biological processes terms in thymoma patients with high and low SOX9 expressions, respectively.

To further explore the potential function of SOX9 in thymoma, we first performed gene set enrichment analysis (GSEA). The top 100 upregulated and downregulated genes identified by GSEA are shown in **Figure 2A**. GSEA results also showed that patients with high SOX9 expression had enrichment for the TGF-β signaling pathway and pathway in cancer (**Figure 2B**), while the low SOX9 expression group had enrichment for the primary immunodeficiency pathway and the T cell receptor signaling pathway (**Supplementary Figure 1**). DEGs related to SOX9 expression were further identified using the *limma* package of the R software (|log_2_(fold-change) | >2; adjusted *P <*0.05). The results revealed that 291 genes were upregulated and 106 genes downregulated in patients with high SOX9 expression compared to those with low expression (**Supplementary Figure 2**). Then, the KEGG pathway and GO enrichment analyses were performed using the *clusterProfiler* R package to investigate the functions of these DEGs. The results from the KEGG pathway enrichment analysis indicated that these 291 upregulated DEGs were mapped to the proteoglycans in cancer, and molecules involved in axon guidance, cell adhesion, extracellular matrix-receptor interaction, and the TGF-β signaling pathway, and that these 106 downregulated DEGs were mapped to molecules involved in primary immunodeficiency, hematopoietic cell lineage, the T cell receptor signaling pathway, Th17 cell differentiation, PD-L1 expression, the PD-1 checkpoint pathway in cancer, Th1 and Th2 cells differentiation, et al. (**Supplementary Figure 3** and **Supplementary Tables 1, 2**). The DEGs related to these pathways are shown in **Figures 2C, D**. The results from the GO enrichment analysis indicated that the upregulated DEGs were mapped to molecules involved in the biological process of mesenchymal cell development, epithelial tube morphogenesis, extracellular matrix organization, stem cell development, et al.; and that the downregulated DEGs were mapped to molecules involved in the immune-related GO terms, such as the antigen receptor-mediated signaling pathway, T cell differentiation in thymus, positive regulation of lymphocyte activation, et al. (**Figures 2E, F** and **Supplementary Tables 3, 4**). Together, these results indicated that SOX9 expression was associated with the epithelial phenotype instead of immune phenotype of thymomas, and we proposed that SOX9 might be used as a potential marker for the epithelial components of thymomas.

### SOX9 Was Correlated With Genes Associated With the Thymic Tuft Cells Phenotype in Thymoma

**Figure 3:**
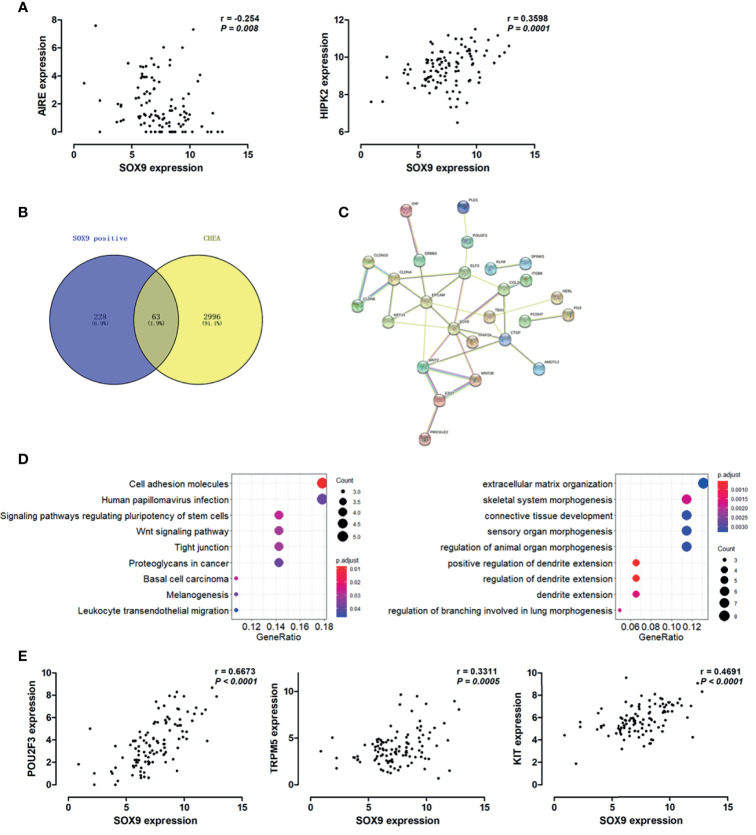
SOX9 expression associated with the thymic tuft cells phenotype of thymomas. **(A)** Scatter plots illustrate the linear regression analyses for the associations between the expression of SOX9 and the expression of HIPK2 and AIRE in thymomas, respectively. **(B)** Venn diagram depicts the 63 common genes which are positively associated with SOX9 expression and regulated by SOX9. **(C)** Protein-protein interaction (PPI) network of these 63 common genes was constructed with the nodes with interaction confidence value > 0.95. Disconnected nodes were hidden in the network. **(D)** The dot-plot depicts the activity of the Kyoto Encyclopedia of Genes and Genomes (KEGG) pathways (right) and the biological processes terms (left) of these 63 genes. **(E)** Scatter plots illustrate the pearson correlation analysis for the associations between the expression of SOX9 and the expression of POU2F3, TRPM5, and KIT in thymomas, respectively.

**Figure 4:**
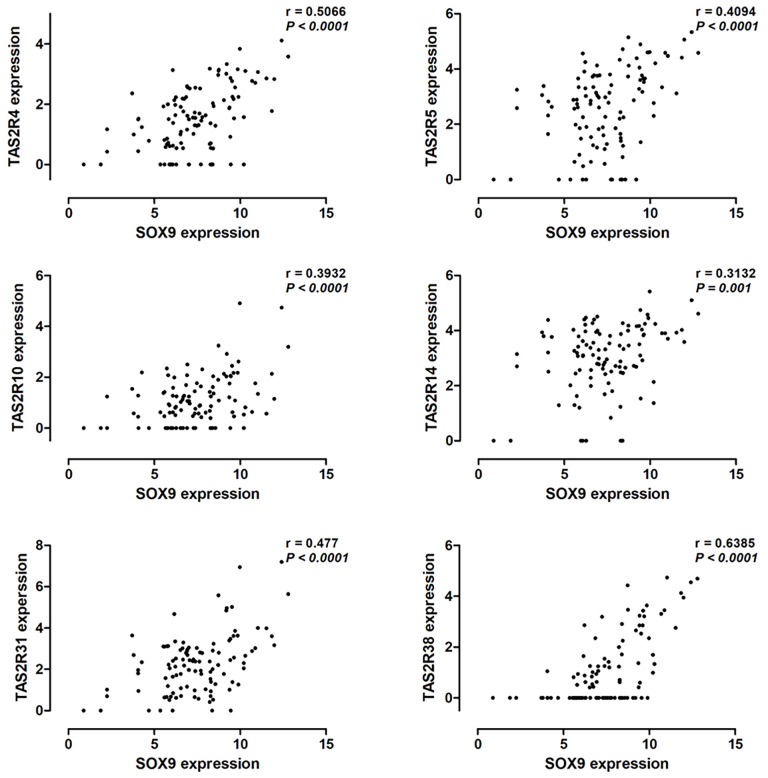
Pearson correlation analysis to explore the correlation between SOX9 expression and the expression of the TAS2R family members.

Hassall’s corpuscles are known as cornified bodies within the medulla of human thymus. As a transcriptional factor, positive nuclear SOX9 staining was observed in the epithelial cells of Hassall’s corpuscles (**Figure 1A**) where thymic tuft cells are located (25). SOX9 has been used as a marker for tuft cells in several tissues (26). These findings suggest a potential role of SOX9 in thymic medullary epithelial cells. Autoimmune regulator (AIRE) and homeodomain-interacting protein kinase 2 (HIPK2) are known as two critical transcriptional factors that play non-redundant roles in determining thymic tuft cells development and shaping thymic function (25, 27). We found that SOX9 expression was negatively correlated with AIRE expression but positively with HIPK2 expression (**Figure 3A**). By crossing the 291 DEGs positively associated with SOX9 expression with genes potentially regulated by SOX9 and identified from the CHEA dataset, 63 genes were identified (**Figure 3B**). The interactions between these 63 genes and SOX9 are shown in **Figure 3C**. The KEGG pathway enrichment analysis indicated that these 63 genes were mapped to molecules involved in the signaling pathway regulating the pluripotency of stem cells, the Wnt signaling pathway, tight junction, et al. (**Figure 3D** and **Supplementary Table 5**). In addition, the GO enrichment analysis revealed that these 63 genes were involved in extracellular matrix organization and sensory organ morphogenesis (**Figure 3D** and **Supplementary Table 6**). Among these genes, *POU2F3*, a transcriptional factor for thymic tuft cells development (25, 28), was shown to be positively correlated with SOX9 expression (**Figure 3E**, *P <*0.001, R^2 =^ 0.445). JASPAR analysis revealed that *POU2F3* carries 6 putative SOX9-binding sites along its DNA transcriptional regulatory region, with a homology higher than 80% (**Supplementary Table 7**). In addition, SOX9 expression was positively associated with the expressions of TRPM5, which is required for the function of thymic tuft cells, and KIT, which is frequently expressed in thymic carcinomas (**Figure 3E**) (25, 28). Moreover, we observed that SOX9 was correlated with almost all members of the TAS2R family (**Figure 4**) which are overexpressed in thymic tuft cells (25). Taken together, these data further supported the notion that SOX9 might be used as a marker for thymic epithelial cells and explained the association of its expression with the histological type of TETs, especially the one representing a tuft cell phenotype of thymomas.

### SOX9 Expression Was Associated With Immune Suppressive Microenvironment of Thymoma

**Figure 5:**
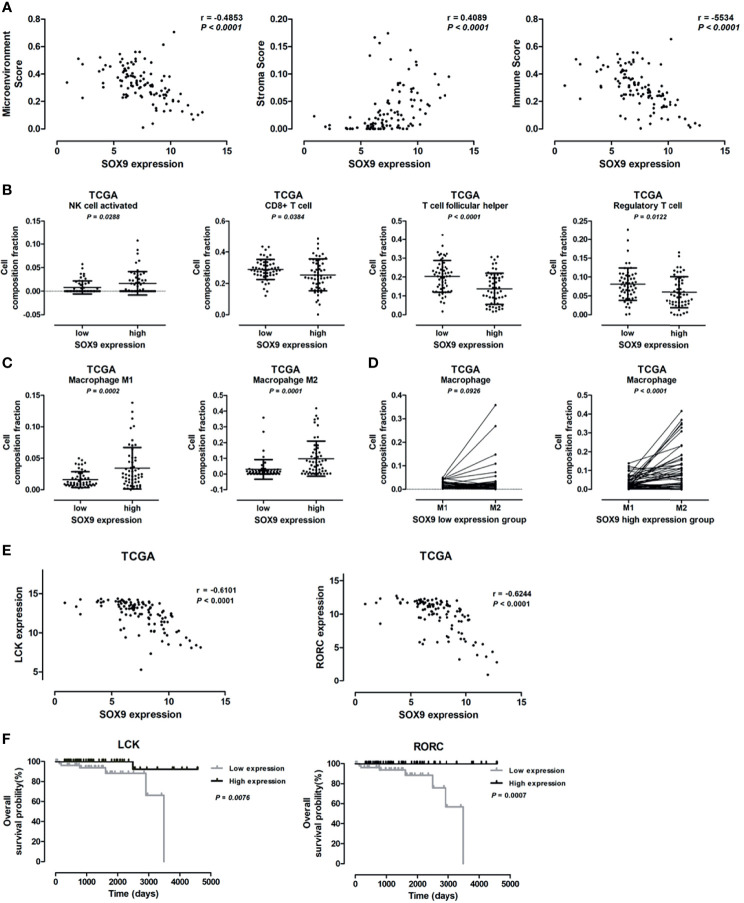
Correlation between SOX9 expression and immune cells infiltration level. **(A)** Scatter plots illustrating the pearson correlation analysis for the associations of SOX9 expression with tumor microenvironment score, stroma score, and immune scores in thymomas, respectively. **(B)** Dot plots representing the infiltration of immune cells, including activated natural killer cell, CD8+ T cell, follicular T cell, and regulatory T cell, between patients with high and low SOX9 expressions. **(C)** Dot plots representing M1 and M2 cells between patients with high and low SOX9 expressions. **(D)** Dot plots representing the infiltration of M1 and M2 cells in patients with high or low SOX9 expression. **(E)** Scatter plots illustrating the pearson correlation analysis for the associations of SOX9 expression with LCK and RORC expression in thymomas, respectively. **(F)** Kaplan–Meier survival curves showing that thymoma patients from TCGA dataset with low LCK or RORC RNA expression had shorter overall survival time (*P* < 0.05), as determined by the log-rank test. Thymoma patients (n = 108) were categorized into high and low LCK or RORC expression groups with the median value of LCK or RORC mRNA expression level as cutoff.

Bioinformatics analysis revealed that SOX9 expression was associated with genes related to the extracellular matrix-receptor interaction pathway and extracellular matrix structure organization, indicating a potential role of SOX9 in regulating tumor microenvironment. We further investigated the role of SOX9 in tumor microenvironment; the microenvironment score, tumor stromal score, and immune score, as well as the infiltration of immune cells including B cells, CD4+T cells, and CD8+T cells in thymoma tumor tissues were analyzed using the TIMER estimation application. In the TIMER estimation, the xCELL and CIBERSORT methods were selected to digitally portray the cellular heterogeneity landscape in tumor tissues. Then, we analyzed the association of SOX9 expression with these scores and immune cells infiltrating levels, and found that SOX9 expression was positively correlated with stromal score but negatively with immune score and microenvironment score (**Figure 5A**). In addition, we found that thymoma patients with higher SOX9 expression had less infiltration of B cells, CD4+ T cells, and CD8+ T cells, but higher infiltration of macrophages. Notably, patients with high SOX9 expression had a significantly higher infiltration of M1 and M2 macrophages compared to the low SOX9 expression group (**Figure 5C**). However, M2 macrophage significantly dominated in the high SOX9 expression group (**Figure 5D**). Of note, we observed that SOX9 expression was negatively associated with the expressions of LCK and RORC, which are involved in the development, function, and differentiation of T and Th17 cells, respectively (**Figure 5E**). Survival analysis revealed that thymoma patients with high LCK or RORC expressions had favorable clinical outcomes (**Figure 5F**). Taken together, we proposed that SOX9 expression might be associated with an immune suppression tumor microenvironment of thymomas.

## Discussion

As a transcription factor, SOX9 has been implicated in the initiation, development, and progression of tumors in multiple organs (15). However, the role of SOX9 in TETs has not been reported yet. In this study, we found that SOX9 was expressed in the nuclei of the epithelial cells of Hassall’s corpuscles and in the epithelial component of TET cells in almost all cases. Of note, SOX9 expression was significantly correlated with the histological type of thymomas and might serve as a negative prognostic marker for thymomas. In addition, bioinformatics analysis further revealed that SOX9 was positively associated with genes regulating epithelial tube morphogenesis and extracellular matrix, and negatively associated with genes related to immune cell differentiation, PD-L1 expression, and the PD-1 checkpoint pathway in cancer. Taken together, these findings suggest that SOX9 could be used as a marker for thymic epithelial cells and a diagnostic and prognostic marker for TETs.

Tuft cells are chemosensory epithelial cells with a unique “tuft” of long and thick microvilli on their apical side (26). Tuft cell-like medullary thymic epithelial cells were identified in murine thymus (25). It has been proposed that thymic tuft cells are immunologically important, highly differentiated epithelial cells in the thymic medulla (25, 28, 29). Yamada et al. reported that a tuft cell-like signature was highly prevalent in thymic squamous cell carcinoma (28). In line with these findings, our results showed that positive nucleus staining of SOX9 was observed in almost all TET tissues. In thymomas, we found that SOX9 expression was positively correlated with the expression of HIPK2, which is a critical transcriptional factor determining thymic tuft cells development and shaping thymic function (25). POU2F3, which is required for the development and function of thymic tuft cells, was found to be highly expressed in thymic squamous cell carcinomas (25, 28). By JASPAR analysis, we found that *POU2F3* might be a target of SOX9. Among the 63 genes potentially regulated by SOX9, 6 genes are involved in sensory organ morphogenesis, including *COL2A1*, *FBN2*, *TBX1*, *TFAP2A*, *WNT2*, and *WNT2B*. In addition, SOX9 was correlated with almost all members of the TAS2R family which is a key component of the canonical taste transduction pathway and may be coordinated in the chemosensory specificities of thymic tuft cells (25). Taken together, our results indicated that SOX9 expression might be associated with the tuft cell phenotype of thymoma.

In this study, we observed that SOX9 expression was associated with the tumor microenvironment (TME) of thymoma, with SOX9 expression positively correlated with the tumor stromal scores while negatively with the tumor immune scores. Bioinformatics analysis revealed that genes positively associated with SOX9 expression in thymomas were enriched in the extracellular matrix-receptor interaction pathway and the TGF-β signaling pathway, the latter of which plays important roles in regulating stromal cells and has potent immunosuppressive effects on both innate and adaptive immune cells in the tumor microenvironment (30). It has been known that immune cells are important constituents of the tumor microenvironment and critically participate in the development and progression of various tumors. Increasing evidence highlights that adaptive immune cells, including T and B cells, and innate immune cells, such as macrophages and natural killer cells, contribute to tumor progression when present in the tumor stroma (31). We observed differential activation of tumor associated macrophages, with patients with high SOX9 expression had enrichment of M2 macrophages. The M2 macrophages, which secrete anti-inflammatory cytokines such as IL-10, IL-13, and IL-4 and which express abundant arginase-1, mannose receptor (MR, CD206), and scavenger receptor, tend to show an immune suppressive phenotype (31, 32). Preciously, it has been revealed that M2-macrophages can suppress the antitumor activity of cytotoxic CD8+ T cells within tumors (33). In line with the previous work, we observed that the number of CD8+ T cells decreased in the tumor samples of patients with high SOX9 expression, while the M2 macrophage abundance increased. Moreover, SOX9 was negatively correlated with genes associated with T or Th17 cell development, such as *LCK* and *RORC*. Survival analysis revealed that thymoma patients with high LCK or RORC had favorable clinical outcomes. Together, these findings suggested that SOX9 expression might indicate a competitive interaction between M2 macrophages and CD8+ T cells, and an immune suppressive microenvironment of thymomas, which consequently led to enhanced pro-tumorigenic activity.

To the best of our knowledge, this is the first study proposing the potential roles of SOX9 in thymoma. However, the precise mechanism of SOX9 in the initiation and progression of TETs were not well investigated in this study, due to a lack of thymic tumor cell lines and the unavailability of animal models of thymic tumors. The bioinformatics findings need to be further validated by both *in vitro* and *in vivo* experiments.

In conclusion, we comprehensively analyzed the expression profile and the diagnostic values of SOX9 in TETs based on immunohistochemistry examination and bioinformatics analysis. Our findings provided evidence that SOX9 could serve as a marker for thymic epithelial cells and as a diagnostic and prognostic marker for TETs. Notably, SOX9 expression in TETs might indicate a tuft cell phenotype and an immune suppressive microenvironment of thymomas. However, the specific role and the precise mechanism of SOX9 in the initiation and progression of TETs need to be further extensively investigated.

## Data Availability Statement

The datasets presented in this study can be found in online repositories. The names of the repository/repositories and accession number(s) can be found in the article/**Supplementary Material**.

## Ethics Statement

The study involving human participants were reviewed and approved by Anhui Medical University and the First Affiliated Hospital of USTC. Written informed consent for participation was not required for this study in accordance with the national legislation and the institutional requirements.

## Author Contributions

Conception, design, and hypothesis: XY, LH, and YL. Clinical design and organization: XY, YL, WL, and YZ. Patients sampling and pathological experiments: WL, XY, and YL. Drafting the article: XY, LH, YL, and FY. Data discussion, reviewing and editing the article critically: XY, YL, LH, BN, and YZ. All authors contributed to the article and approved the submitted version.

## Funding

This study was supported by the Fundamental Research Funds for the Central Universities (grant number WK9110000131 to XY) and National Natural Science Foundation of China (grant number 32000492 to FY).

## Conflict of Interest

The authors declare that the research was conducted in the absence of any commercial or financial relationships that could be construed as a potential conflict of interest.

## Publisher’s Note

All claims expressed in this article are solely those of the authors and do not necessarily represent those of their affiliated organizations, or those of the publisher, the editors and the reviewers. Any product that may be evaluated in this article, or claim that may be made by its manufacturer, is not guaranteed or endorsed by the publisher.

## Supplementary Material

Supplementary Figure 1 GSEA analysis revealed the enrichment of the gene sets related to the “pathway in cancer and TGF-β signaling pathway” in patients with high SOX9 expression.

Supplementary Figure 2 Volcano plot showing the differentially up- and down-regulated genes in thymoma patients with high SOX9 expression compared to those with low SOX9 expression. The median value of SOX9 expression level was selected as cutoff. Differentially expressed genes (DEGs) satisfying the criteria of |log_2_(fold-change) | >2 with an adjusted *P <*0.05 was considered as significant. DEGs positively and negatively correlated with SOX9 expression are represented as red and green dots, respectively.

Supplementary Figure 3 The dot-plot depicts the activity of the KEGG pathways in thymoma patients with high and low SOX9 expressions, respectively.

## Abbreviations

BP, biological process; CC, cellular component; DEG, differentially expressed gene; GO, gene ontology; GSEA, Gene Set Enrichment Analysis; KEGG, Kyoto Encyclopedia of Genes and Genomes; MF, molecular function; OS, overall survival; PPI, Protein–Protein Interaction; SD, standard deviation; SOX9, SRY-related high-mobility group box 9; TCGA, The Cancer Genome Atlas; TETs, thymic epithelial tumors; TIMER, Tumor Immune Estimation Resource; TICs, Tumor-Infiltrating Immune Cells; TME, tumor microenvironment; WHO, World Health Organization.
