# Supplementary figures and images for: Diagnostic and Prognostic Significances of SOX9 in Thymic Epithelial Tumor
Source: Front Oncol. 2021 Oct 28;11:708735. doi: 10.3389/fonc.2021.708735 (PMC8580949; doi:10.3389/fonc.2021.708735)

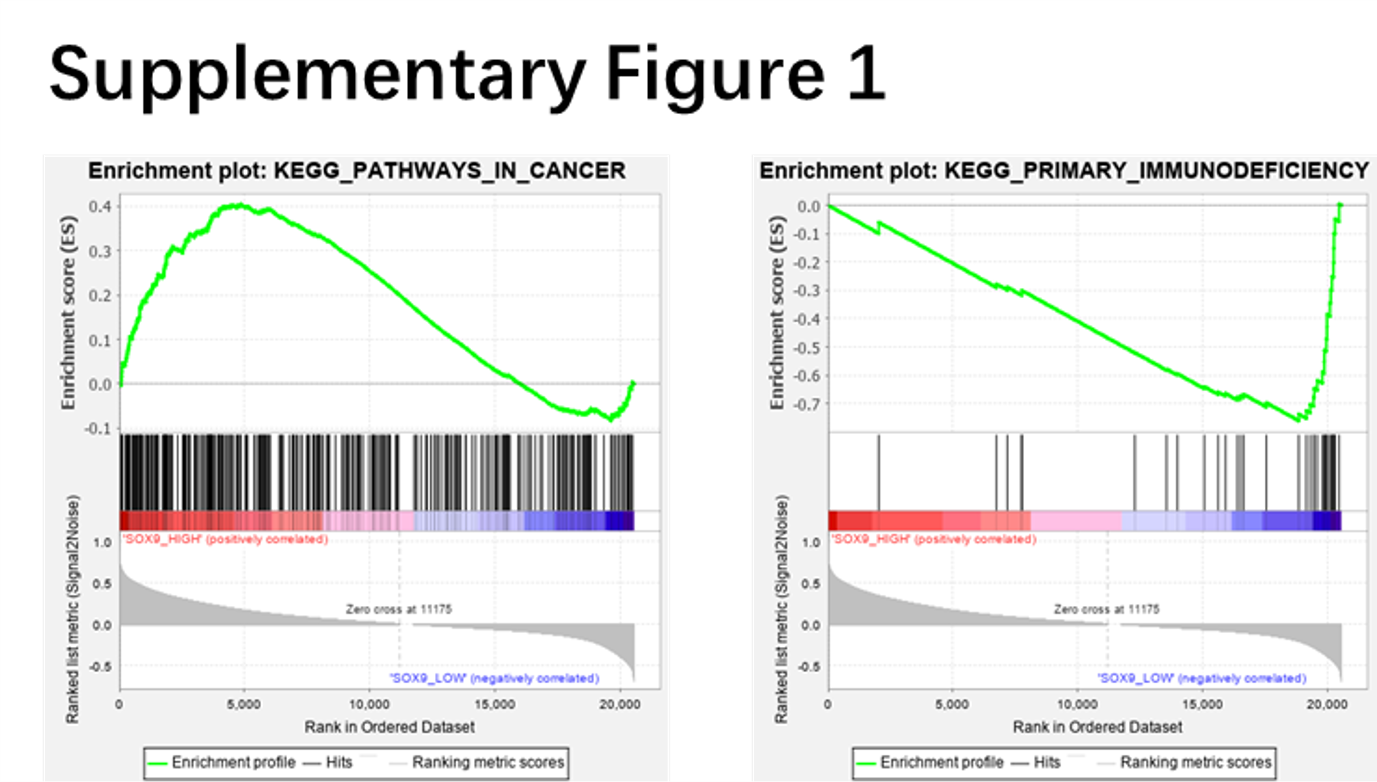

Supplement: Supplementary Figure 1 — GSEA analysis revealed the enrichment of the gene sets related to the “pathway in cancer and TGF-β signaling pathway” in patients with high SOX9 expression. [file Image_1.tiff]

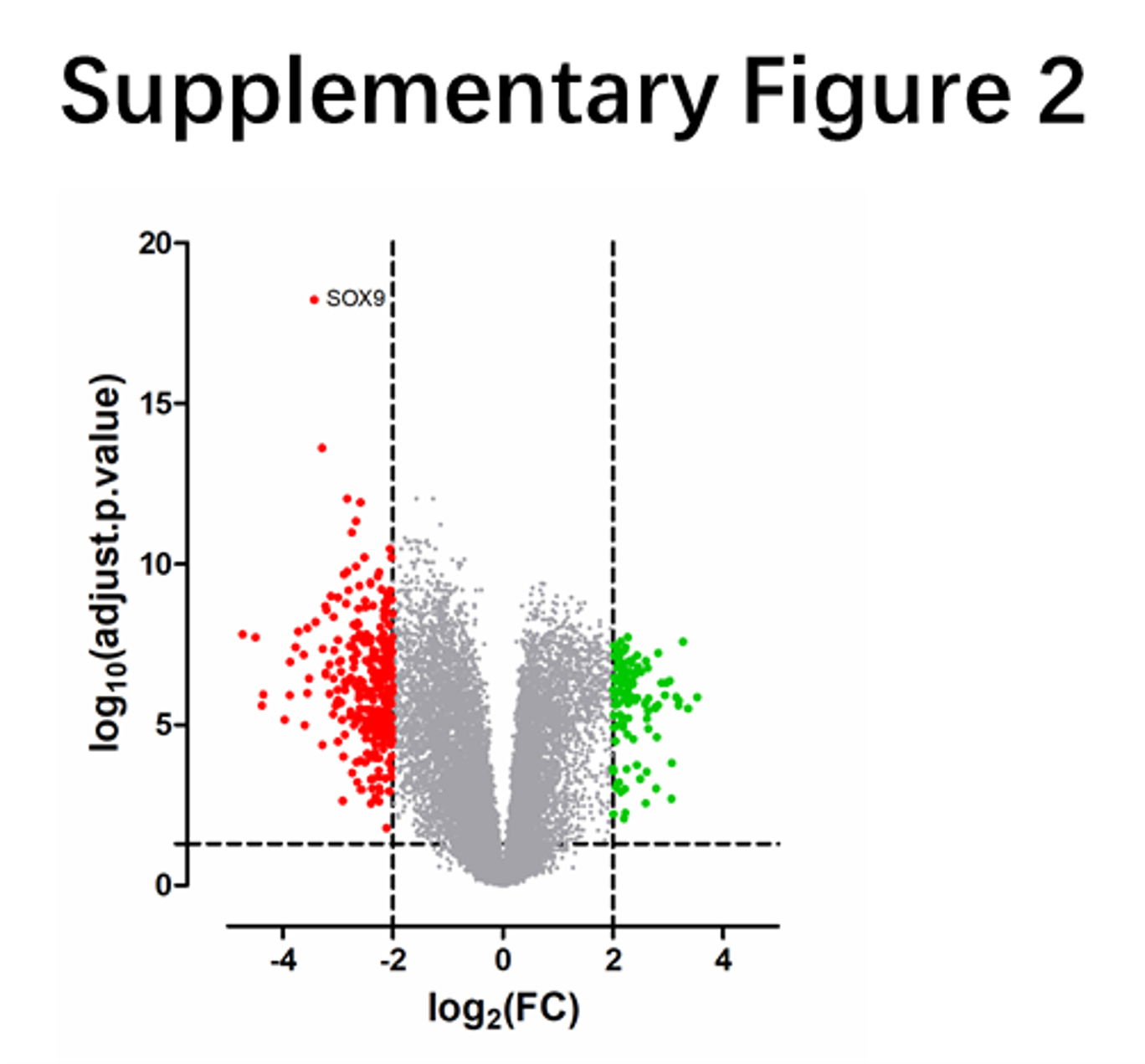

Supplement: Supplementary Figure 2 — Volcano plot showing the differentially up- and down-regulated genes in thymoma patients with high SOX9 expression compared to those with low SOX9 expression. The median value of SOX9 expression level was selected as cutoff. Differentially expressed genes (DEGs) satisfying the criteria of |log2(fold-change) | >2 with an adjusted P <0.05 was considered as significant. DEGs positively and negatively correlated with SOX9 expression are represented as red and green dots, respectively. [file Image_2.tiff]

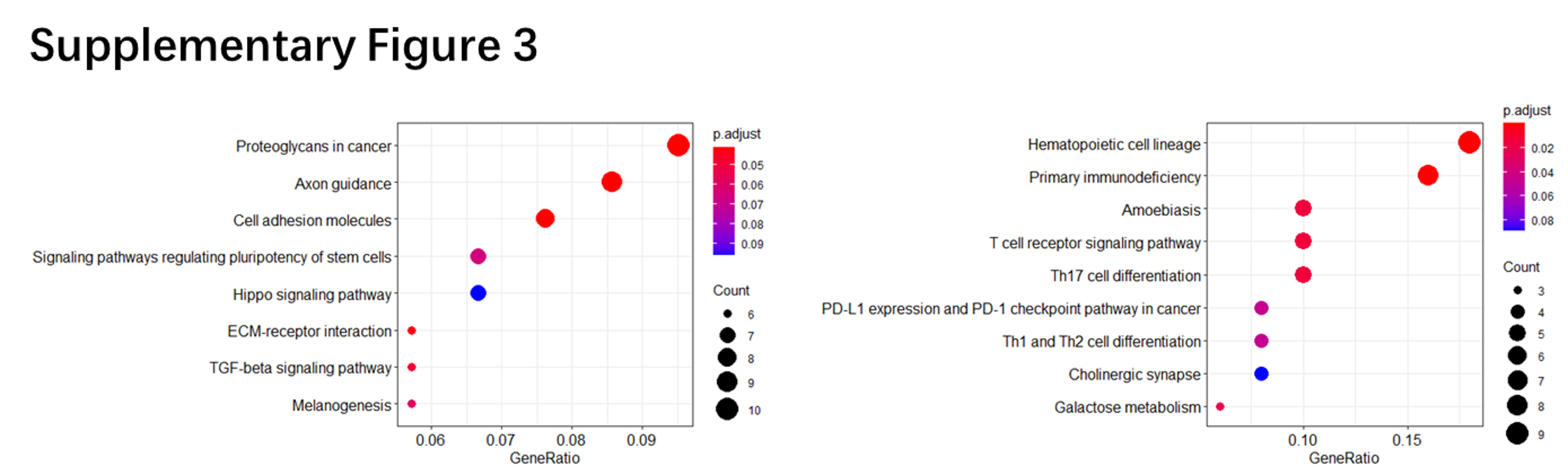

Supplement: Supplementary Figure 3 — The dot-plot depicts the activity of the KEGG pathways in thymoma patients with high and low SOX9 expressions, respectively. [file Image_3.tiff]
