# Supplementary material for: Diagnostic and Prognostic Significances of SOX9 in Thymic Epithelial Tumor
Source: Front Oncol. 2021 Oct 28;11:708735. doi: 10.3389/fonc.2021.708735 (PMC8580949; doi:10.3389/fonc.2021.708735)
Supplement: Supplementary file 4 [file Table_1.docx]

Supplementary Table 1. Kyoto Encyclopedia of Genes and Genomes (KEGG) pathway enrichment analysis of 291 genes were upregulated in patients with high SOX9 expression

| ID | Description | Gene Ratio | *P* value | Adjusted P value | q value | Gene | Count |
| --- | --- | --- | --- | --- | --- | --- | --- |
| a05205 | Proteoglycans in cancer | 10/105 | 0.000314 | 0.04115 | 0.038291 | WNT2/SDC4/ERBB4/ERBB3/FN1/WNT2B/FZD7/MET/FLNC/WNT5B | 10 |
| hsa04360 | Axon guidance | 9/105 | 0.000573 | 0.04115 | 0.038291 | SEMA3D/SEMA3E/NGEF/MET/NTN1/SLIT2/MYL9/WNT5B/EPHA3 | 9 |
| hsa04514 | Cell adhesion molecules | 8/105 | 0.000682 | 0.04115 | 0.038291 | CLDN8/ITGB8/SDC4/CNTN1/CLDN10/CLDN3/IGSF11/CLDN4 | 8 |
| hsa04512 | ECM-receptor interaction | 6/105 | 0.000954 | 0.043183 | 0.040181 | ITGB8/COL9A3/SDC4/FN1/COL2A1/FRAS1 | 6 |
| hsa04350 | TGF-β signaling pathway | 6/105 | 0.001347 | 0.048751 | 0.045363 | THSD4/ID4/GDF5/FST/SMAD9/BMP8B | 6 |
| hsa04916 | Melanogenesis | 6/105 | 0.001949 | 0.058783 | 0.054698 | WNT2/TYRP1/WNT2B/FZD7/WNT5B/ADCY5 | 6 |
| hsa04550 | Signaling pathways regulating pluripotency of stem cells | 7/105 | 0.00252 | 0.06516 | 0.060632 | WNT2/ID4/WNT2B/FZD7/SMAD9/SOX2/WNT5B | 7 |
| hsa04390 | Hippo signaling pathway | 7/105 | 0.004238 | 0.095879 | 0.089216 | WNT2/WNT2B/GDF5/FZD7/SOX2/WNT5B/BMP8B | 7 |

ECM, extracellular matrix; TGF, tumor growth factor.

Supplementary Table 2. Kyoto Encyclopedia of Genes and Genomes (KEGG) pathway enrichment analysis of 106 genes downregulated in patients with high SOX9 expression

| ID | Description | Gene Ratio | *P* value | Adjusted P value | q value | Gene ID | Count |
| --- | --- | --- | --- | --- | --- | --- | --- |
| hsa05340 | Primary immunodeficiency | 8/50 | 5.08E-11 | 7.98E-09 | 6.85E-09 | CD79A/LCK/IGLL1/RAG1/CD3E/ADA/CD3D/RAG2 | 8 |
| hsa04640 | Hematopoietic cell lineage | 9/50 | 7.11E-09 | 5.58E-07 | 4.79E-07 | MME/CD3E/CD1C/CD38/CD3D/CD1A/CD1E/CD1B/DNTT | 9 |
| hsa05146 | Amoebiasis | 5/50 | 0.000392 | 0.015351 | 0.013175 | CD1C/PRKCG/CD1A/CD1E/CD1B | 5 |
| hsa04660 | T cell receptor signaling pathway | 5/50 | 0.000429 | 0.015351 | 0.013175 | LCK/CD247/CD3E/GRAP2/CD3D | 5 |
| hsa04659 | Th17 cell differentiation | 5/50 | 0.000489 | 0.015351 | 0.013175 | LCK/CD247/CD3E/RORC/CD3D | 5 |
| hsa00052 | Galactose metabolism | 3/50 | 0.000887 | 0.023207 | 0.019916 | AKR1B10/LCT/HKDC1 | 3 |
| hsa05235 | PD-L1 expression and PD-1 checkpoint pathway in cancer | 4/50 | 0.002152 | 0.047668 | 0.040909 | LCK/CD247/CD3E/CD3D | 4 |
| hsa04658 | Th1 and Th2 cell differentiation | 4/50 | 0.002429 | 0.047668 | 0.040909 | LCK/CD247/CD3E/CD3D | 4 |
| hsa04725 | Cholinergic synapse | 4/50 | 0.00509 | 0.088796 | 0.076204 | CAMK4/KCNJ4/PRKCG/CHRNA3 | 4 |
