# Supplementary material for: Diagnostic and Prognostic Significances of SOX9 in Thymic Epithelial Tumor
Source: Front Oncol. 2021 Oct 28;11:708735. doi: 10.3389/fonc.2021.708735 (PMC8580949; doi:10.3389/fonc.2021.708735)
Supplement: Supplementary file 5 [file Table_2.docx]

Supplementary Table 3. Gene Ontology (GO) analysis of 291 genes were upregulated in patients with high SOX9 expression

| Ontology | ID | Description | Gene Ratio | *P* value | Adjusted *P* value | q value | Gene ID | Count |
| --- | --- | --- | --- | --- | --- | --- | --- | --- |
| BP | GO:0098742 | cell-cell adhesion via plasma-membrane adhesion molecules | 17/253 | 9.55E-08 | 0.000168788 | 0.000145263 | CLDN8/PCDHGA12/PCDHGB7/PCDH7/CNTN4/CLDN10/DCHS2/DCHS2/PCDHGA11/CLDN3/SLITRK2/IGSF11/PCDHGB2/PCDH15/CLDN4/FAT1/EPCAM | 17 |
| BP | GO:0030198 | extracellular matrix organization | 20/253 | 2.15E-07 | 0.000168788 | 0.000145263 | SOX9/FBN2/ITGB8/THSD4/COL9A3/ADAMTS20/COL11A1/KLK7/MMP3/FN1/SPINK5/SCUBE1/SCUBE3/FLRT2/MMP13/COL2A1/ICAM5/COL28A1/ELF3/ADAMTS5 | 20 |
| BP | GO:0043062 | extracellular structure organization | 20/253 | 2.23E-07 | 0.000168788 | 0.000145263 | SOX9/FBN2/ITGB8/THSD4/COL9A3/ADAMTS20/COL11A1/KLK7/MMP3/FN1/SPINK5/SCUBE1/SCUBE3/FLRT2/MMP13/COL2A1/ICAM5/COL28A1/ELF3/ADAMTS5 | 20 |
| BP | GO:0045665 | negative regulation of neuron differentiation | 15/253 | 2.56E-07 | 0.000168788 | 0.000145263 | KLK8/SEMA3D/SOX9/SEMA3E/NGEF/ID4/NGFR/CNTN4/IRX3/ZNF536/NTN1/PTPRG/SLIT2/SOX2/LRP4 | 15 |
| BP | GO:0001755 | neural crest cell migration | 8/253 | 5.35E-07 | 0.000262107 | 0.000225576 | TBX1/SEMA3D/SEMA3E/ERBB4/FN1/FOLR1/EDN3/GBX2 | 8 |
| BP | GO:0090596 | sensory organ morphogenesis | 16/253 | 5.96E-07 | 0.000262107 | 0.000225576 | AQP5/TBX1/SOX9/FBN2/WNT2/TULP1/TFAP2A/COL11A1/SALL1/WNT2B/COL2A1/ALDH1A3/TSPAN12/GBX2/NTN1/FAT1 | 16 |
| BP | GO:0014032 | neural crest cell development | 9/253 | 7.59E-07 | 0.000286106 | 0.000246231 | TBX1/SEMA3D/SOX9/SEMA3E/ERBB4/FN1/FOLR1/EDN3/GBX2 | 9 |
| BP | GO:0014031 | mesenchymal cell development | 9/253 | 1.14E-06 | 0.000369196 | 0.00031774 | TBX1/SEMA3D/SOX9/SEMA3E/ERBB4/FN1/FOLR1/EDN3/GBX2 | 9 |
| BP | GO:0048864 | stem cell development | 9/253 | 1.26E-06 | 0.000369196 | 0.00031774 | TBX1/SEMA3D/SOX9/SEMA3E/ERBB4/FN1/FOLR1/EDN3/GBX2 | 9 |
| BP | GO:0014033 | neural crest cell differentiation | 9/253 | 2.02E-06 | 0.000505509 | 0.000435055 | TBX1/SEMA3D/SOX9/SEMA3E/ERBB4/FN1/FOLR1/EDN3/GBX2 | 9 |
| BP | GO:0050768 | negative regulation of neurogenesis | 16/253 | 2.11E-06 | 0.000505509 | 0.000435055 | KLK8/SEMA3D/SOX9/SEMA3E/NGEF/ERBB4/ID4/NGFR/CNTN4/IRX3/ZNF536/NTN1/PTPRG/SLIT2/SOX2/LRP4 | 16 |
| BP | GO:0060562 | epithelial tube morphogenesis | 17/253 | 3.13E-06 | 0.000687603 | 0.000591769 | IRX2/TBX1/SOX9/SEMA3E/WNT2/SDC4/SALL1/WNT2B/FOLR1/IRX3/TACSTD2/LRP2/MET/GBX2/SALL4/NTN1/SLIT2 | 17 |
| BP | GO:0010721 | negative regulation of cell development | 17/253 | 4.18E-06 | 0.000821599 | 0.000707091 | KLK8/SEMA3D/SOX9/SEMA3E/NGEF/ERBB4/ID4/NGFR/CNTN4/IRX3/TACSTD2/ZNF536/NTN1/PTPRG/SLIT2/SOX2/LRP4 | 17 |
| BP | GO:0016331 | morphogenesis of embryonic epithelium | 11/253 | 4.36E-06 | 0.000821599 | 0.000707091 | IRX2/SOX9/WNT2/TFAP2A/SDC4/WNT2B/FOLR1/IRX3/ALDH1A3/LRP2/SALL4 | 11 |
| BP | GO:0051961 | negative regulation of nervous system development | 16/253 | 4.99E-06 | 0.000877796 | 0.000755455 | KLK8/SEMA3D/SOX9/SEMA3E/NGEF/ERBB4/ID4/NGFR/CNTN4/IRX3/ZNF536/NTN1/PTPRG/SLIT2/SOX2/LRP4 | 16 |
| BP | GO:0048762 | mesenchymal cell differentiation | 13/253 | 5.95E-06 | 0.000980573 | 0.000843908 | TBX1/SEMA3D/SOX9/SEMA3E/WNT2/ERBB4/FN1/FOLR1/EDN3/VASN/VASN/GBX2/EPHA3 | 13 |
| BP | GO:0042476 | odontogenesis | 10/253 | 7.86E-06 | 0.001218939 | 0.001049052 | AQP5/TBX1/PHEX/SP6/TFAP2A/AQP6/WDR72/EDAR/LRP4/ADAMTS5 | 10 |
| BP | GO:0043583 | ear development | 13/253 | 9.42E-06 | 0.00132477 | 0.001140133 | TBX1/SOX9/TFAP2A/SDC4/COL11A1/SALL1/ATP8B1/COL2A1/ALDH1A3/GBX2/PCDH15/NTN1/SOX2 | 13 |
| BP | GO:0007156 | homophilic cell adhesion via plasma membrane adhesion molecules | 11/253 | 9.83E-06 | 0.00132477 | 0.001140133 | PCDHGA12/PCDHGB7/PCDH7/CNTN4/DCHS2/DCHS2/PCDHGA11/IGSF11/PCDHGB2/PCDH15/FAT1 | 11 |
| BP | GO:0048839 | inner ear development | 12/253 | 1.00E-05 | 0.00132477 | 0.001140133 | TBX1/SOX9/TFAP2A/SDC4/COL11A1/ATP8B1/COL2A1/ALDH1A3/GBX2/PCDH15/NTN1/SOX2 | 12 |
| BP | GO:0007409 | axonogenesis | 19/253 | 1.30E-05 | 0.001629425 | 0.001402328 | SEMA3D/SLITRK5/SEMA3E/GFRA3/NGFR/FN1/FLRT2/CNTN4/PTPRZ1/GBX2/SLITRK2/ARTN/NTN1/SLIT2/LRP4/KIF5C/KIF5C/EPHA3/GRB7 | 19 |
| BP | GO:0072073 | kidney epithelium development | 10/253 | 1.38E-05 | 0.001650301 | 0.001420294 | IRX2/SOX9/CRLF1/SDC4/SALL1/WNT2B/IRX3/TACSTD2/SLIT2/EPCAM | 10 |
| BP | GO:0060485 | mesenchyme development | 14/253 | 1.58E-05 | 0.001808384 | 0.001556344 | TBX1/SEMA3D/SOX9/SEMA3E/WNT2/ERBB4/ERBB3/FN1/FOLR1/EDN3/VASN/VASN/GBX2/EPHA3 | 14 |
| BP | GO:0051216 | cartilage development | 12/253 | 1.86E-05 | 0.0020385 | 0.001754388 | SOX9/ITGB8/SCARA3/COL11A1/WNT2B/GDF5/MMP13/VWA2/COL2A1/CHI3L1/WNT5B/BMP8B | 12 |
| BP | GO:0042471 | ear morphogenesis | 9/253 | 3.02E-05 | 0.003188617 | 0.002744211 | TBX1/SOX9/TFAP2A/COL11A1/SALL1/COL2A1/ALDH1A3/GBX2/NTN1 | 9 |
| BP | GO:0001657 | ureteric bud development | 8/253 | 3.58E-05 | 0.00349321 | 0.003006351 | SOX9/CRLF1/SDC4/SALL1/WNT2B/TACSTD2/SLIT2/EPCAM | 8 |
| BP | GO:0150063 | visual system development | 16/253 | 3.63E-05 | 0.00349321 | 0.003006351 | AQP5/SOX9/FBN2/WNT2/TULP1/TFAP2A/WNT2B/CRYAB/MAB21L2/CPAMD8/C3/ALDH1A3/TSPAN12/SOX2/WNT5B/FAT1 | 16 |
| BP | GO:0072163 | mesonephric epithelium development | 8/253 | 3.84E-05 | 0.00349321 | 0.003006351 | SOX9/CRLF1/SDC4/SALL1/WNT2B/TACSTD2/SLIT2/EPCAM | 8 |
| BP | GO:0072164 | mesonephric tubule development | 8/253 | 3.84E-05 | 0.00349321 | 0.003006351 | SOX9/CRLF1/SDC4/SALL1/WNT2B/TACSTD2/SLIT2/EPCAM | 8 |
| BP | GO:0010771 | negative regulation of cell morphogenesis involved in differentiation | 8/253 | 4.73E-05 | 0.004137282 | 0.003560657 | SEMA3D/SEMA3E/NGEF/NGFR/TACSTD2/NTN1/SLIT2/LRP4 | 8 |
| BP | GO:0001823 | mesonephros development | 8/253 | 5.06E-05 | 0.004137282 | 0.003560657 | SOX9/CRLF1/SDC4/SALL1/WNT2B/TACSTD2/SLIT2/EPCAM | 8 |
| BP | GO:0048592 | eye morphogenesis | 10/253 | 5.10E-05 | 0.004137282 | 0.003560657 | AQP5/SOX9/FBN2/WNT2/TULP1/TFAP2A/WNT2B/ALDH1A3/TSPAN12/FAT1 | 10 |
| BP | GO:0048880 | sensory system development | 16/253 | 5.18E-05 | 0.004137282 | 0.003560657 | AQP5/SOX9/FBN2/WNT2/TULP1/TFAP2A/WNT2B/CRYAB/MAB21L2/CPAMD8/C3/ALDH1A3/TSPAN12/SOX2/WNT5B/FAT1 | 16 |
| BP | GO:0061448 | connective tissue development | 13/253 | 5.49E-05 | 0.004257074 | 0.003663753 | SOX9/ITGB8/SCARA3/COL11A1/ID4/WNT2B/GDF5/MMP13/VWA2/COL2A1/CHI3L1/WNT5B/BMP8B | 13 |
| BP | GO:0042472 | inner ear morphogenesis | 8/253 | 5.79E-05 | 0.004361008 | 0.003753202 | TBX1/SOX9/TFAP2A/COL11A1/COL2A1/ALDH1A3/GBX2/NTN1 | 8 |
| BP | GO:0007411 | axon guidance | 13/253 | 6.33E-05 | 0.004503645 | 0.003875959 | SEMA3D/SEMA3E/GFRA3/FLRT2/CNTN4/GBX2/ARTN/NTN1/SLIT2/KIF5C/KIF5C/EPHA3/GRB7 | 13 |
| BP | GO:0019731 | antibacterial humoral response | 6/253 | 6.39E-05 | 0.004503645 | 0.003875959 | SLPI/KLK7/SPINK5/RNASE7/DEFB1/DEFB1 | 6 |
| BP | GO:0097485 | neuron projection guidance | 13/253 | 6.56E-05 | 0.004503645 | 0.003875959 | SEMA3D/SEMA3E/GFRA3/FLRT2/CNTN4/GBX2/ARTN/NTN1/SLIT2/KIF5C/KIF5C/EPHA3/GRB7 | 13 |
| BP | GO:0071772 | response to BMP | 10/253 | 6.83E-05 | 0.004503645 | 0.003875959 | SOX9/GDF5/FST/TDGF1/COL2A1/SMAD9/MYH6/LRP2/GDF1/BMP8B | 10 |
| BP | GO:0071773 | cellular response to BMP stimulus | 10/253 | 6.83E-05 | 0.004503645 | 0.003875959 | SOX9/GDF5/FST/TDGF1/COL2A1/SMAD9/MYH6/LRP2/GDF1/BMP8B | 10 |
| BP | GO:0001822 | kidney development | 13/253 | 8.35E-05 | 0.005369364 | 0.00462102 | IRX2/SOX9/CRLF1/TFAP2A/SDC4/SALL1/WNT2B/IRX3/TACSTD2/SLIT2/LRP4/FRAS1/EPCAM | 13 |
| BP | GO:0001667 | ameboidal-type cell migration | 17/253 | 0.000110672 | 0.006948599 | 0.005980153 | TBX1/SEMA3D/SOX9/SEMA3E/SDC4/ERBB4/FN1/AMOTL2/TDGF1/FOLR1/EDN3/TACSTD2/MET/GBX2/PTPRG/SLIT2/EPPK1 | 17 |
| BP | GO:0001654 | eye development | 15/253 | 0.000116577 | 0.007024064 | 0.0060451 | AQP5/SOX9/FBN2/WNT2/TULP1/TFAP2A/WNT2B/CRYAB/MAB21L2/CPAMD8/ALDH1A3/TSPAN12/SOX2/WNT5B/FAT1 | 15 |
| BP | GO:0001655 | urogenital system development | 14/253 | 0.000117201 | 0.007024064 | 0.0060451 | IRX2/SOX9/CRLF1/TFAP2A/SDC4/ID4/SALL1/WNT2B/IRX3/TACSTD2/SLIT2/LRP4/FRAS1/EPCAM | 14 |
| BP | GO:0072001 | renal system development | 13/253 | 0.000136697 | 0.008010459 | 0.006894018 | IRX2/SOX9/CRLF1/TFAP2A/SDC4/SALL1/WNT2B/IRX3/TACSTD2/SLIT2/LRP4/FRAS1/EPCAM | 13 |
| BP | GO:0031345 | negative regulation of cell projection organization | 10/253 | 0.000159445 | 0.009070048 | 0.007805929 | KLK8/SEMA3D/SEMA3E/NGEF/NGFR/TACSTD2/NTN1/PTPRG/SLIT2/LRP4 | 10 |
| BP | GO:0002062 | chondrocyte differentiation | 8/253 | 0.000161658 | 0.009070048 | 0.007805929 | SOX9/SCARA3/COL11A1/WNT2B/GDF5/VWA2/COL2A1/WNT5B | 8 |
| BP | GO:0035107 | appendage morphogenesis | 9/253 | 0.000169903 | 0.009143565 | 0.0078692 | SOX9/FBN2/TFAP2A/SALL1/GDF5/COL2A1/SALL4/LRP4/FRAS1 | 9 |
| BP | GO:0035108 | limb morphogenesis | 9/253 | 0.000169903 | 0.009143565 | 0.0078692 | SOX9/FBN2/TFAP2A/SALL1/GDF5/COL2A1/SALL4/LRP4/FRAS1 | 9 |
| CC | GO:0062023 | collagen-containing extracellular matrix | 22/261 | 1.96E-08 | 5.28E-06 | 4.23E-06 | FBN2/WNT2/THSD4/SLPI/SCARA3/COL9A3/ADAMTS20/COL11A1/BCAM/FN1/WNT2B/EDIL3/VWA2/COL2A1/PTPRZ1/F3/COL28A1/NTN1/CLU/WNT5B/FRAS1/ADAMTS5 | 22 |
| CC | GO:0016324 | apical plasma membrane | 18/261 | 3.19E-07 | 4.30E-05 | 3.45E-05 | AQP5/SLC34A2/AQP6/ERBB3/FN1/ATP8B1/AMOTL2/TDGF1/SHANK2/FOLR1/SCNN1G/LRP2/ANO1/P2RX2/CLDN4/PLD1/FAT1/EPCAM | 18 |
| CC | GO:0045177 | apical part of cell | 19/261 | 1.10E-06 | 9.83E-05 | 7.88E-05 | AQP5/SLC34A2/AQP6/ERBB3/FN1/ATP8B1/AMOTL2/EDAR/TDGF1/SHANK2/FOLR1/SCNN1G/LRP2/ANO1/P2RX2/CLDN4/PLD1/FAT1/EPCAM | 19 |
| CC | GO:0045178 | basal part of cell | 7/261 | 6.06E-06 | 0.00040757 | 0.000326949 | AQP5/ERBB4/ERBB3/TACSTD2/MET/KRT14/CLDN4 | 7 |
| CC | GO:0009925 | basal plasma membrane | 6/261 | 9.13E-06 | 0.000490991 | 0.000393869 | AQP5/ERBB4/ERBB3/TACSTD2/MET/CLDN4 | 6 |
| CC | GO:0016323 | basolateral plasma membrane | 12/261 | 4.18E-05 | 0.001876108 | 0.001504997 | AQP5/CLDN8/ERBB4/ERBB3/CHRM3/FOLR1/TACSTD2/MET/CLDN4/SLC4A11/EPPK1/EPCAM | 12 |
| CC | GO:0016327 | apicolateral plasma membrane | 4/261 | 6.93E-05 | 0.002661656 | 0.002135157 | CLDN8/CLDN3/CLDN4/EPPK1 | 4 |
| CC | GO:0005581 | collagen trimer | 7/261 | 0.000133521 | 0.004489629 | 0.003601541 | C1QTNF9B/FCN2/SCARA3/COL9A3/COL11A1/COL2A1/COL28A1 | 7 |
| CC | GO:0005923 | bicellular tight junction | 8/261 | 0.00020318 | 0.006072838 | 0.004871578 | CLDN8/AMOTL2/CLDN10/TACSTD2/CLDN3/CLDN4/EPPK1/EPCAM | 8 |
| CC | GO:0070160 | tight junction | 8/261 | 0.000261927 | 0.007045834 | 0.005652107 | CLDN8/AMOTL2/CLDN10/TACSTD2/CLDN3/CLDN4/EPPK1/EPCAM | 8 |
| CC | GO:0005911 | cell-cell junction | 15/261 | 0.000387339 | 0.009472203 | 0.007598519 | CLDN8/SGCA/GJB7/FLRT2/AMOTL2/PCDHGA12/CLDN10/TACSTD2/CLDN3/GJD3/IGSF11/CLDN4/FAT1/EPPK1/EPCAM | 15 |
| MF | GO:0005201 | extracellular matrix structural constituent | 11/247 | 2.68E-05 | 0.007955843 | 0.006659298 | FBN3/FBN2/THSD4/COL9A3/COL11A1/FN1/EDIL3/COL2A1/CHI3L1/COL28A1/FRAS1 | 11 |
| MF | GO:0008236 | serine-type peptidase activity | 11/247 | 6.46E-05 | 0.007955843 | 0.006659298 | KLK8/KLK10/KLK7/MMP3/PRSS12/PRSS22/PRSS22/SCPEP1/F3/KLK11/TMPRSS13 | 11 |
| MF | GO:0016825 | hydrolase activity, acting on acid phosphorus-nitrogen bonds | 11/247 | 7.67E-05 | 0.007955843 | 0.006659298 | KLK8/KLK10/KLK7/MMP3/PRSS12/PRSS22/PRSS22/SCPEP1/F3/KLK11/TMPRSS13 | 11 |
| MF | GO:0017171 | serine hydrolase activity | 11/247 | 7.67E-05 | 0.007955843 | 0.006659298 | KLK8/KLK10/KLK7/MMP3/PRSS12/PRSS22/PRSS22/SCPEP1/F3/KLK11/TMPRSS13 | 11 |
| MF | GO:0004252 | serine-type endopeptidase activity | 10/247 | 0.000120503 | 0.00842712 | 0.007053772 | KLK8/KLK10/KLK7/MMP3/PRSS12/PRSS22/PRSS22/F3/KLK11/TMPRSS13 | 10 |
| MF | GO:0043394 | proteoglycan binding | 5/247 | 0.000121838 | 0.00842712 | 0.007053772 | FCN2/FN1/FST/COL2A1/SLIT2 | 5 |

Supplementary Table 4. Gene Ontology (GO) analysis of 106 genes downregulated in patients with high SOX9 expression

| Ontology | ID | Description | GeneRatio | P value | Adjusted *P* value | q value | Gene | Count |
| --- | --- | --- | --- | --- | --- | --- | --- | --- |
| BP | GO:0030217 | T cell differentiation | 12/90 | 1.95E-09 | 3.20E-06 | 2.80E-06 | LCK/CAMK4/RAG1/CD3E/TCF7/MYB/LEF1/ADA/RORC/CD3D/CCR9/RAG2 | 12 |
| BP | GO:0030098 | lymphocyte differentiation | 13/90 | 1.21E-08 | 9.95E-06 | 8.72E-06 | CD79A/LCK/CAMK4/RAG1/CD3E/TCF7/MYB/LEF1/ADA/RORC/CD3D/CCR9/RAG2 | 13 |
| BP | GO:0033151 | V(D)J recombination | 4/90 | 4.54E-07 | 0.000248 | 0.000218 | RAG1/TCF7/LEF1/RAG2 | 4 |
| BP | GO:0033077 | T cell differentiation in thymus | 6/90 | 8.73E-07 | 0.000358 | 0.000314 | CAMK4/RAG1/CD3E/ADA/CD3D/RAG2 | 6 |
| BP | GO:0050851 | antigen receptor-mediated signaling pathway | 10/90 | 2.73E-06 | 0.000898 | 0.000787 | CD79A/LCK/IGLL1/CD247/TRAT1/CD3E/GRAP2/CD38/ADA/CD3D | 10 |
| BP | GO:0070229 | negative regulation of lymphocyte apoptotic process | 4/90 | 9.91E-06 | 0.002715 | 0.002377 | AURKB/RAG1/ADA/PTCRA | 4 |
| BP | GO:0051251 | positive regulation of lymphocyte activation | 9/90 | 1.63E-05 | 0.003224 | 0.002823 | LCK/IGLL1/RAG1/CD3E/GRAP2/CD38/MYB/ADA/TMIGD2 | 9 |
| BP | GO:0070243 | regulation of thymocyte apoptotic process | 3/90 | 1.74E-05 | 0.003224 | 0.002823 | RAG1/ADA/PTCRA | 3 |
| BP | GO:0031343 | positive regulation of cell killing | 6/90 | 1.77E-05 | 0.003224 | 0.002823 | CD1C/CD5L/CD1A/SH2D1A/CD1E/CD1B | 6 |
| BP | GO:0050862 | positive regulation of T cell receptor signaling pathway | 3/90 | 2.86E-05 | 0.004701 | 0.004117 | LCK/TRAT1/ADA | 3 |
| BP | GO:0002696 | positive regulation of leukocyte activation | 9/90 | 5.03E-05 | 0.007151 | 0.006263 | LCK/IGLL1/RAG1/CD3E/GRAP2/CD38/MYB/ADA/TMIGD2 | 9 |
| BP | GO:0070242 | thymocyte apoptotic process | 3/90 | 5.29E-05 | 0.007151 | 0.006263 | RAG1/ADA/PTCRA | 3 |
| BP | GO:0050863 | regulation of T cell activation | 9/90 | 5.66E-05 | 0.007151 | 0.006263 | LCK/CAMK4/RAG1/CD3E/SIT1/GRAP2/MYB/ADA/TMIGD2 | 9 |
| BP | GO:0042100 | B cell proliferation | 5/90 | 7.47E-05 | 0.007888 | 0.006908 | CD79A/CD38/LEF1/ADA/RAG2 | 5 |
| BP | GO:0070233 | negative regulation of T cell apoptotic process | 3/90 | 7.50E-05 | 0.007888 | 0.006908 | RAG1/ADA/PTCRA | 3 |
| BP | GO:0050867 | positive regulation of cell activation | 9/90 | 7.68E-05 | 0.007888 | 0.006908 | LCK/IGLL1/RAG1/CD3E/GRAP2/CD38/MYB/ADA/TMIGD2 | 9 |
| BP | GO:0046632 | alpha-beta T cell differentiation | 5/90 | 8.22E-05 | 0.007944 | 0.006958 | TCF7/MYB/LEF1/ADA/RORC | 5 |
| BP | GO:0046631 | alpha-beta T cell activation | 6/90 | 9.06E-05 | 0.007978 | 0.006987 | CD3E/TCF7/MYB/LEF1/ADA/RORC | 6 |
| BP | GO:2000107 | negative regulation of leukocyte apoptotic process | 4/90 | 9.23E-05 | 0.007978 | 0.006987 | AURKB/RAG1/ADA/PTCRA | 4 |
| BP | GO:0042113 | B cell activation | 7/90 | 0.000112 | 0.008725 | 0.007641 | CD79A/IGLL1/RAG1/CD38/LEF1/ADA/RAG2 | 7 |
| BP | GO:0031295 | T cell costimulation | 4/90 | 0.000114 | 0.008725 | 0.007641 | LCK/CD3E/GRAP2/TMIGD2 | 4 |
| BP | GO:0031294 | lymphocyte costimulation | 4/90 | 0.000122 | 0.008725 | 0.007641 | LCK/CD3E/GRAP2/TMIGD2 | 4 |
| BP | GO:0070228 | regulation of lymphocyte apoptotic process | 4/90 | 0.000122 | 0.008725 | 0.007641 | AURKB/RAG1/ADA/PTCRA | 4 |
| BP | GO:0001912 | positive regulation of leukocyte mediated cytotoxicity | 5/90 | 0.000134 | 0.009164 | 0.008026 | CD1C/CD1A/SH2D1A/CD1E/CD1B | 5 |
| BP | GO:0002475 | antigen processing and presentation via MHC class Ib | 4/90 | 0.000139 | 0.009164 | 0.008026 | CD1C/CD1A/CD1E/CD1B | 4 |
| BP | GO:0050870 | positive regulation of T cell activation | 7/90 | 0.000158 | 0.009819 | 0.008599 | LCK/RAG1/CD3E/GRAP2/MYB/ADA/TMIGD2 | 7 |
| BP | GO:0050857 | positive regulation of antigen receptor-mediated signaling pathway | 3/90 | 0.000175 | 0.009819 | 0.008599 | LCK/TRAT1/ADA | 3 |
| BP | GO:0001916 | positive regulation of T cell mediated cytotoxicity | 4/90 | 0.000179 | 0.009819 | 0.008599 | CD1C/CD1A/CD1E/CD1B | 4 |
| BP | GO:0002562 | somatic diversification of immune receptors via germline recombination within a single locus | 4/90 | 0.000179 | 0.009819 | 0.008599 | RAG1/TCF7/LEF1/RAG2 | 4 |
| BP | GO:0016444 | somatic cell DNA recombination | 4/90 | 0.000179 | 0.009819 | 0.008599 | RAG1/TCF7/LEF1/RAG2 | 4 |
| CC | GO:0042101 | T cell receptor complex | 4/93 | 7.26E-07 | 0.000125 | 0.000108 | CD247/TRAT1/CD3E/CD3D | 4 |
| CC | GO:0009897 | external side of plasma membrane | 11/93 | 2.30E-06 | 0.000198 | 0.000171 | CD79A/IGLL1/CD3E/ENPP3/CD1C/ADA/CD3D/CD1A/CD1E/CCR9/CD1B | 11 |
| CC | GO:0098802 | plasma membrane signaling receptor complex | 7/93 | 2.34E-05 | 0.001342 | 0.001158 | CD79A/CD247/TRAT1/CD3E/CHRNA2/CD3D/CHRNA3 | 7 |
| MF | GO:0071723 | lipopeptide binding | 4/91 | 8.81E-08 | 2.33E-05 | 1.91E-05 | CD1C/CD1A/CD1E/CD1B | 4 |
