# Supplementary material for: Diagnostic and Prognostic Significances of SOX9 in Thymic Epithelial Tumor
Source: Front Oncol. 2021 Oct 28;11:708735. doi: 10.3389/fonc.2021.708735 (PMC8580949; doi:10.3389/fonc.2021.708735)
Supplement: Supplementary file 6 [file Table_3.docx]

Supplementary Table 5. Kyoto Encyclopedia of Genes and Genomes (KEGG) pathway enrichment analysis of 63 genes potentially regulated by transcriptional factor SOX9

| ID | Description | *P* value | Adjusted *P* value | q value | Gene ID | Count |
| --- | --- | --- | --- | --- | --- | --- |
| hsa04514 | Cell adhesion molecules | 0.000137 | 0.008106 | 0.005785 | CLDN10/CLDN4/CLDN8/IGSF11/ITGB8 | 5 |
| hsa05217 | Basal cell carcinoma | 0.001278 | 0.026951 | 0.019234 | FZD7/WNT2/WNT2B | 3 |
| hsa04550 | Signaling pathways regulating pluripotency of stem cells | 0.00137 | 0.026951 | 0.019234 | FZD7/ID4/WNT2/WNT2B | 4 |
| hsa04310 | Wnt signaling pathway | 0.00237 | 0.029853 | 0.021305 | FZD7/PRICKLE2/WNT2/WNT2B | 4 |
| hsa04530 | Tight junction | 0.00253 | 0.029853 | 0.021305 | AMOTL2/CLDN10/CLDN4/CLDN8 | 4 |
| hsa04916 | Melanogenesis | 0.004913 | 0.037324 | 0.026637 | FZD7/WNT2/WNT2B | 3 |
| hsa05165 | Human papillomavirus infection | 0.004997 | 0.037324 | 0.026637 | COL2A1/FZD7/ITGB8/WNT2/WNT2B | 5 |
| hsa05205 | Proteoglycans in cancer | 0.005061 | 0.037324 | 0.026637 | ERBB3/FZD7/WNT2/WNT2B | 4 |
| hsa04670 | Leukocyte transendothelial migration | 0.00688 | 0.045101 | 0.032186 | CLDN10/CLDN4/CLDN8 | 3 |
