# Supplementary material for: Diagnostic and Prognostic Significances of SOX9 in Thymic Epithelial Tumor
Source: Front Oncol. 2021 Oct 28;11:708735. doi: 10.3389/fonc.2021.708735 (PMC8580949; doi:10.3389/fonc.2021.708735)
Supplement: Supplementary file 7 [file Table_4.docx]

Supplementary Table 6. Gene Ontology (GO) analysis of 63 genes potentially regulated by transcriptional factor SOX9

| Ontology | ID | Description | *P* value | Adjusted *P* value | q value | Gene ID | Count |
| --- | --- | --- | --- | --- | --- | --- | --- |
| BP | GO:1903861 | positive regulation of dendrite extension | 7.16E-07 | 0.000566 | 0.000406 | CPNE6/CPNE6/SYT1/SYT17 | 4 |
| BP | GO:1903859 | regulation of dendrite extension | 1.00E-06 | 0.000566 | 0.000406 | CPNE6/CPNE6/SYT1/SYT17 | 4 |
| BP | GO:0097484 | dendrite extension | 4.32E-06 | 0.001626 | 0.001168 | CPNE6/CPNE6/SYT1/SYT17 | 4 |
| BP | GO:0061046 | regulation of branching involved in lung morphogenesis | 6.98E-06 | 0.001748 | 0.001256 | SOX9/WNT2/WNT2B | 3 |
| BP | GO:0048705 | skeletal system morphogenesis | 7.75E-06 | 0.001748 | 0.001256 | SOX9/COL2A1/FBN2/MDFI/TBX1/TFAP2A/VWA2 | 7 |
| BP | GO:0061448 | connective tissue development | 2.07E-05 | 0.003193 | 0.002295 | SOX9/CHI3L1/COL2A1/ID4/ITGB8/VWA2/WNT2B | 7 |
| BP | GO:0090596 | sensory organ morphogenesis | 2.21E-05 | 0.003193 | 0.002295 | SOX9/COL2A1/FBN2/TBX1/TFAP2A/WNT2/WNT2B | 7 |
| BP | GO:2000027 | regulation of animal organ morphogenesis | 2.26E-05 | 0.003193 | 0.002295 | SOX9/FZD7/PRICKLE2/TBX1/TFAP2A/WNT2/WNT2B | 7 |
| BP | GO:0030198 | extracellular matrix organization | 2.85E-05 | 0.003271 | 0.00235 | SOX9/ADAMTS20/COL2A1/ELF3/FBN2/ICAM5/ITGB8/SPINK5 | 8 |
| BP | GO:0043062 | extracellular structure organization | 2.90E-05 | 0.003271 | 0.00235 | SOX9/ADAMTS20/COL2A1/ELF3/FBN2/ICAM5/ITGB8/SPINK5 | 8 |
| BP | GO:0051216 | cartilage development | 4.68E-05 | 0.004411 | 0.003169 | SOX9/CHI3L1/COL2A1/ITGB8/VWA2/WNT2B | 6 |
| BP | GO:0042476 | odontogenesis | 5.41E-05 | 0.004411 | 0.003169 | AQP6/LRP4/TBX1/TFAP2A/WDR72 | 5 |
| BP | GO:0002053 | positive regulation of mesenchymal cell proliferation | 5.47E-05 | 0.004411 | 0.003169 | SOX9/TBX1/WNT2 | 3 |
| BP | GO:0016338 | calcium-independent cell-cell adhesion via plasma membrane cell-adhesion molecules | 5.47E-05 | 0.004411 | 0.003169 | CLDN10/CLDN4/CLDN8 | 3 |
| BP | GO:0034505 | tooth mineralization | 6.93E-05 | 0.004697 | 0.003375 | TBX1/TFAP2A/WDR72 | 3 |
| BP | GO:0071300 | cellular response to retinoic acid | 6.94E-05 | 0.004697 | 0.003375 | SOX9/FZD7/TBX1/WNT2 | 4 |
| BP | GO:0045665 | negative regulation of neuron differentiation | 7.08E-05 | 0.004697 | 0.003375 | SOX9/ID4/KLK8/LRP4/PTPRG/SEMA3E | 6 |
| BP | GO:0001655 | urogenital system development | 7.65E-05 | 0.004793 | 0.003444 | SOX9/EPCAM/ID4/IRX2/LRP4/TFAP2A/WNT2B | 7 |
| BP | GO:0110110 | positive regulation of animal organ morphogenesis | 0.000104 | 0.005533 | 0.003976 | SOX9/TFAP2A/WNT2/WNT2B | 4 |
| BP | GO:0060560 | developmental growth involved in morphogenesis | 0.000106 | 0.005533 | 0.003976 | SOX9/CPNE6/CPNE6/SEMA3E/SYT1/SYT17 | 6 |
| BP | GO:0016331 | morphogenesis of embryonic epithelium | 0.000106 | 0.005533 | 0.003976 | SOX9/IRX2/TFAP2A/WNT2/WNT2B | 5 |
| BP | GO:0035107 | appendage morphogenesis | 0.000113 | 0.005533 | 0.003976 | SOX9/COL2A1/FBN2/LRP4/TFAP2A | 5 |
| BP | GO:0035108 | limb morphogenesis | 0.000113 | 0.005533 | 0.003976 | SOX9/COL2A1/FBN2/LRP4/TFAP2A | 5 |
| BP | GO:0048588 | developmental cell growth | 0.000118 | 0.005545 | 0.003985 | SOX9/CPNE6/CPNE6/SEMA3E/SYT1/SYT17 | 6 |
| BP | GO:0010464 | regulation of mesenchymal cell proliferation | 0.000128 | 0.005761 | 0.00414 | SOX9/TBX1/WNT2 | 3 |
| BP | GO:0071277 | cellular response to calcium ion | 0.000136 | 0.005915 | 0.00425 | CPNE6/CPNE6/SYT1/SYT17 | 4 |
| BP | GO:0048592 | eye morphogenesis | 0.00015 | 0.006081 | 0.004369 | SOX9/FBN2/TFAP2A/WNT2/WNT2B | 5 |
| BP | GO:0031128 | developmental induction | 0.000166 | 0.006081 | 0.004369 | SOX9/WNT2/WNT2B | 3 |
| BP | GO:1905332 | positive regulation of morphogenesis of an epithelium | 0.000166 | 0.006081 | 0.004369 | SOX9/WNT2/WNT2B | 3 |
| BP | GO:0031214 | biomineral tissue development | 0.000167 | 0.006081 | 0.004369 | SOX9/FBN2/TBX1/TFAP2A/WDR72 | 5 |
| BP | GO:0110148 | biomineralization | 0.000167 | 0.006081 | 0.004369 | SOX9/FBN2/TBX1/TFAP2A/WDR72 | 5 |
| BP | GO:0060441 | epithelial tube branching involved in lung morphogenesis | 0.00018 | 0.006356 | 0.004567 | SOX9/WNT2/WNT2B | 3 |
| BP | GO:0048704 | embryonic skeletal system morphogenesis | 0.000191 | 0.006458 | 0.004641 | COL2A1/MDFI/TBX1/TFAP2A | 4 |
| BP | GO:0098742 | cell-cell adhesion via plasma-membrane adhesion molecules | 0.000195 | 0.006458 | 0.004641 | CLDN10/CLDN4/CLDN8/EPCAM/IGSF11/PCDH7 | 6 |
| BP | GO:1990138 | neuron projection extension | 0.000211 | 0.00681 | 0.004893 | CPNE6/CPNE6/SEMA3E/SYT1/SYT17 | 5 |
| BP | GO:0001822 | kidney development | 0.000243 | 0.007623 | 0.005478 | SOX9/EPCAM/IRX2/LRP4/TFAP2A/WNT2B | 6 |
| BP | GO:0048736 | appendage development | 0.00027 | 0.008024 | 0.005765 | SOX9/COL2A1/FBN2/LRP4/TFAP2A | 5 |
| BP | GO:0060173 | limb development | 0.00027 | 0.008024 | 0.005765 | SOX9/COL2A1/FBN2/LRP4/TFAP2A | 5 |
| BP | GO:0048562 | embryonic organ morphogenesis | 0.000291 | 0.008331 | 0.005986 | SOX9/COL2A1/FBN2/MDFI/TBX1/TFAP2A | 6 |
| BP | GO:0042472 | inner ear morphogenesis | 0.0003 | 0.008331 | 0.005986 | SOX9/COL2A1/TBX1/TFAP2A | 4 |
| BP | GO:0010463 | mesenchymal cell proliferation | 0.000303 | 0.008331 | 0.005986 | SOX9/TBX1/WNT2 | 3 |
| BP | GO:0072001 | renal system development | 0.000317 | 0.008475 | 0.00609 | SOX9/EPCAM/IRX2/LRP4/TFAP2A/WNT2B | 6 |
| BP | GO:0071248 | cellular response to metal ion | 0.000326 | 0.008475 | 0.00609 | CPNE6/CPNE6/SYT1/SYT17/TFAP2A | 5 |
| BP | GO:0048639 | positive regulation of developmental growth | 0.000333 | 0.008475 | 0.00609 | CPNE6/CPNE6/SYT1/SYT17/WNT2 | 5 |
| BP | GO:0050768 | negative regulation of neurogenesis | 0.000345 | 0.008475 | 0.00609 | SOX9/ID4/KLK8/LRP4/PTPRG/SEMA3E | 6 |
| BP | GO:0060351 | cartilage development involved in endochondral bone morphogenesis | 0.000346 | 0.008475 | 0.00609 | SOX9/COL2A1/VWA2 | 3 |
| BP | GO:0060349 | bone morphogenesis | 0.000368 | 0.00884 | 0.006352 | SOX9/COL2A1/TFAP2A/VWA2 | 4 |
| BP | GO:0048568 | embryonic organ development | 0.000449 | 0.010111 | 0.007265 | SOX9/COL2A1/FBN2/MDFI/TBX1/TFAP2A/WNT2 | 7 |
| BP | GO:0021545 | cranial nerve development | 0.000469 | 0.010111 | 0.007265 | ERBB3/TBX1/TFAP2A | 3 |
| BP | GO:2001054 | negative regulation of mesenchymal cell apoptotic process | 0.000469 | 0.010111 | 0.007265 | SOX9/TBX1 | 2 |
| BP | GO:1905330 | regulation of morphogenesis of an epithelium | 0.000472 | 0.010111 | 0.007265 | SOX9/FZD7/PRICKLE2/WNT2/WNT2B | 5 |
| BP | GO:0042303 | molting cycle | 0.000476 | 0.010111 | 0.007265 | SOX9/KRT14/LRP4/SPINK5 | 4 |
| BP | GO:0042633 | hair cycle | 0.000476 | 0.010111 | 0.007265 | SOX9/KRT14/LRP4/SPINK5 | 4 |
| BP | GO:0007389 | pattern specification process | 0.00049 | 0.010111 | 0.007265 | IRX2/IRX4/LRP4/MDFI/TBX1/WNT2/WNT2B | 7 |
| BP | GO:0051961 | negative regulation of nervous system development | 0.000493 | 0.010111 | 0.007265 | SOX9/ID4/KLK8/LRP4/PTPRG/SEMA3E | 6 |
| BP | GO:0002062 | chondrocyte differentiation | 0.000522 | 0.010517 | 0.007557 | SOX9/COL2A1/VWA2/WNT2B | 4 |
| BP | GO:0032526 | response to retinoic acid | 0.000538 | 0.010647 | 0.007651 | SOX9/FZD7/TBX1/WNT2 | 4 |
| BP | GO:0042471 | ear morphogenesis | 0.000571 | 0.011103 | 0.007978 | SOX9/COL2A1/TBX1/TFAP2A | 4 |
| BP | GO:0071241 | cellular response to inorganic substance | 0.000591 | 0.011133 | 0.008 | CPNE6/CPNE6/SYT1/SYT17/TFAP2A | 5 |
| BP | GO:0043010 | camera-type eye development | 0.000592 | 0.011133 | 0.008 | SOX9/CRYAB/FBN2/TFAP2A/WNT2/WNT2B | 6 |
| BP | GO:0048706 | embryonic skeletal system development | 0.000623 | 0.011519 | 0.008277 | COL2A1/MDFI/TBX1/TFAP2A | 4 |
| BP | GO:0060174 | limb bud formation | 0.000663 | 0.011868 | 0.008528 | SOX9/COL2A1 | 2 |
| BP | GO:2001053 | regulation of mesenchymal cell apoptotic process | 0.000663 | 0.011868 | 0.008528 | SOX9/TBX1 | 2 |
| BP | GO:0060425 | lung morphogenesis | 0.000685 | 0.012073 | 0.008675 | SOX9/WNT2/WNT2B | 3 |
| BP | GO:0060562 | epithelial tube morphogenesis | 0.000737 | 0.012796 | 0.009194 | SOX9/IRX2/SEMA3E/TBX1/WNT2/WNT2B | 6 |
| BP | GO:0097152 | mesenchymal cell apoptotic process | 0.000772 | 0.013096 | 0.00941 | SOX9/TBX1 | 2 |
| BP | GO:0048593 | camera-type eye morphogenesis | 0.000778 | 0.013096 | 0.00941 | SOX9/TFAP2A/WNT2/WNT2B | 4 |
| BP | GO:0048638 | regulation of developmental growth | 0.000791 | 0.013128 | 0.009433 | CPNE6/CPNE6/SEMA3E/SYT1/SYT17/WNT2 | 6 |
| BP | GO:0010721 | negative regulation of cell development | 0.000825 | 0.013491 | 0.009694 | SOX9/ID4/KLK8/LRP4/PTPRG/SEMA3E | 6 |
| BP | GO:0003002 | regionalization | 0.000872 | 0.014054 | 0.010099 | IRX2/LRP4/MDFI/TBX1/WNT2/WNT2B | 6 |
| BP | GO:0071599 | otic vesicle development | 0.000889 | 0.014122 | 0.010147 | SOX9/COL2A1 | 2 |
| BP | GO:0072073 | kidney epithelium development | 0.000934 | 0.014636 | 0.010517 | SOX9/EPCAM/IRX2/WNT2B | 4 |
| BP | GO:0010038 | response to metal ion | 0.000959 | 0.014821 | 0.01065 | CPNE6/CPNE6/KRT14/SYT1/SYT17/TFAP2A | 6 |
| BP | GO:0060572 | morphogenesis of an epithelial bud | 0.001014 | 0.015456 | 0.011106 | WNT2/WNT2B | 2 |
| BP | GO:0001654 | eye development | 0.001081 | 0.016254 | 0.011679 | SOX9/CRYAB/FBN2/TFAP2A/WNT2/WNT2B | 6 |
| BP | GO:0060688 | regulation of morphogenesis of a branching structure | 0.001136 | 0.016586 | 0.011918 | SOX9/WNT2/WNT2B | 3 |
| BP | GO:0150063 | visual system development | 0.001138 | 0.016586 | 0.011918 | SOX9/CRYAB/FBN2/TFAP2A/WNT2/WNT2B | 6 |
| BP | GO:0021783 | preganglionic parasympathetic fiber development | 0.001147 | 0.016586 | 0.011918 | TBX1/TFAP2A | 2 |
| BP | GO:0051592 | response to calcium ion | 0.001166 | 0.016653 | 0.011966 | CPNE6/CPNE6/SYT1/SYT17 | 4 |
| BP | GO:0060350 | endochondral bone morphogenesis | 0.001285 | 0.018125 | 0.013024 | SOX9/COL2A1/VWA2 | 3 |
| BP | GO:0048880 | sensory system development | 0.001326 | 0.018462 | 0.013266 | SOX9/CRYAB/FBN2/TFAP2A/WNT2/WNT2B | 6 |
| BP | GO:0010977 | negative regulation of neuron projection development | 0.001404 | 0.019291 | 0.013862 | KLK8/LRP4/PTPRG/SEMA3E | 4 |
| BP | GO:0048486 | parasympathetic nervous system development | 0.001437 | 0.019291 | 0.013862 | TBX1/TFAP2A | 2 |
| BP | GO:0090190 | positive regulation of branching involved in ureteric bud morphogenesis | 0.001437 | 0.019291 | 0.013862 | SOX9/WNT2B | 2 |
| BP | GO:0001502 | cartilage condensation | 0.001593 | 0.020623 | 0.014819 | SOX9/COL2A1 | 2 |
| BP | GO:0070166 | enamel mineralization | 0.001593 | 0.020623 | 0.014819 | TBX1/WDR72 | 2 |
| BP | GO:0021675 | nerve development | 0.00168 | 0.020623 | 0.014819 | ERBB3/TBX1/TFAP2A | 3 |
| BP | GO:0072078 | nephron tubule morphogenesis | 0.00168 | 0.020623 | 0.014819 | SOX9/IRX2/WNT2B | 3 |
| BP | GO:0060485 | mesenchyme development | 0.001692 | 0.020623 | 0.014819 | SOX9/ERBB3/SEMA3E/TBX1/WNT2 | 5 |
| BP | GO:0001503 | ossification | 0.001709 | 0.020623 | 0.014819 | SOX9/COL2A1/FBN2/ID4/LRP4/TFAP2A | 6 |
| BP | GO:0016202 | regulation of striated muscle tissue development | 0.00171 | 0.020623 | 0.014819 | ERBB3/FZD7/TBX1/WNT2 | 4 |
| BP | GO:0030903 | notochord development | 0.001758 | 0.020623 | 0.014819 | SOX9/COL2A1 | 2 |
| BP | GO:0072079 | nephron tubule formation | 0.001758 | 0.020623 | 0.014819 | SOX9/IRX2 | 2 |
| BP | GO:0072088 | nephron epithelium morphogenesis | 0.001804 | 0.020623 | 0.014819 | SOX9/IRX2/WNT2B | 3 |
| BP | GO:1901861 | regulation of muscle tissue development | 0.001822 | 0.020623 | 0.014819 | ERBB3/FZD7/TBX1/WNT2 | 4 |
| BP | GO:0060537 | muscle tissue development | 0.001833 | 0.020623 | 0.014819 | SOX9/ERBB3/FZD7/NEBL/TBX1/WNT2 | 6 |
| BP | GO:0048634 | regulation of muscle organ development | 0.00186 | 0.020623 | 0.014819 | ERBB3/FZD7/TBX1/WNT2 | 4 |
| BP | GO:0014032 | neural crest cell development | 0.001869 | 0.020623 | 0.014819 | SOX9/SEMA3E/TBX1 | 3 |
| BP | GO:0045165 | cell fate commitment | 0.001906 | 0.020623 | 0.014819 | SOX9/FZD7/TBX1/WNT2/WNT2B | 5 |
| BP | GO:0045927 | positive regulation of growth | 0.001906 | 0.020623 | 0.014819 | CPNE6/CPNE6/SYT1/SYT17/WNT2 | 5 |
| BP | GO:0001759 | organ induction | 0.00193 | 0.020623 | 0.014819 | WNT2/WNT2B | 2 |
| BP | GO:0061213 | positive regulation of mesonephros development | 0.00193 | 0.020623 | 0.014819 | SOX9/WNT2B | 2 |
| BP | GO:0002088 | lens development in camera-type eye | 0.001935 | 0.020623 | 0.014819 | CRYAB/WNT2/WNT2B | 3 |
| BP | GO:0061333 | renal tubule morphogenesis | 0.001935 | 0.020623 | 0.014819 | SOX9/IRX2/WNT2B | 3 |
| BP | GO:0072028 | nephron morphogenesis | 0.001935 | 0.020623 | 0.014819 | SOX9/IRX2/WNT2B | 3 |
| BP | GO:0048754 | branching morphogenesis of an epithelial tube | 0.001938 | 0.020623 | 0.014819 | SOX9/SEMA3E/WNT2/WNT2B | 4 |
| BP | GO:0030500 | regulation of bone mineralization | 0.002003 | 0.021111 | 0.015169 | SOX9/FBN2/TFAP2A | 3 |
| BP | GO:0048608 | reproductive structure development | 0.002031 | 0.021217 | 0.015246 | SOX9/ID4/ITGB8/MDFI/WNT2/WNT2B | 6 |
| BP | GO:0061458 | reproductive system development | 0.002101 | 0.021435 | 0.015402 | SOX9/ID4/ITGB8/MDFI/WNT2/WNT2B | 6 |
| BP | GO:0048791 | calcium ion-regulated exocytosis of neurotransmitter | 0.002109 | 0.021435 | 0.015402 | SYT1/SYT17 | 2 |
| BP | GO:0098743 | cell aggregation | 0.002109 | 0.021435 | 0.015402 | SOX9/COL2A1 | 2 |
| BP | GO:0014031 | mesenchymal cell development | 0.002142 | 0.021571 | 0.0155 | SOX9/SEMA3E/TBX1 | 3 |
| BP | GO:0048864 | stem cell development | 0.002214 | 0.022097 | 0.015878 | SOX9/SEMA3E/TBX1 | 3 |
| BP | GO:0090103 | cochlea morphogenesis | 0.002297 | 0.022527 | 0.016187 | SOX9/TBX1 | 2 |
| BP | GO:0090189 | regulation of branching involved in ureteric bud morphogenesis | 0.002297 | 0.022527 | 0.016187 | SOX9/WNT2B | 2 |
| BP | GO:0050673 | epithelial cell proliferation | 0.002346 | 0.022786 | 0.016373 | SOX9/EHF/FZD7/KLK8/TBX1/WNT2 | 6 |
| BP | GO:0030324 | lung development | 0.002363 | 0.022786 | 0.016373 | SOX9/CHI3L1/WNT2/WNT2B | 4 |
| BP | GO:0060571 | morphogenesis of an epithelial fold | 0.002492 | 0.023818 | 0.017115 | WNT2/WNT2B | 2 |
| BP | GO:0030323 | respiratory tube development | 0.00255 | 0.024174 | 0.01737 | SOX9/CHI3L1/WNT2/WNT2B | 4 |
| BP | GO:0014033 | neural crest cell differentiation | 0.002595 | 0.024337 | 0.017487 | SOX9/SEMA3E/TBX1 | 3 |
| BP | GO:0030307 | positive regulation of cell growth | 0.002647 | 0.024337 | 0.017487 | CPNE6/CPNE6/SYT1/SYT17 | 4 |
| BP | GO:0001558 | regulation of cell growth | 0.002668 | 0.024337 | 0.017487 | CPNE6/CPNE6/CRYAB/SEMA3E/SYT1/SYT17 | 6 |
| BP | GO:0001942 | hair follicle development | 0.002676 | 0.024337 | 0.017487 | SOX9/LRP4/SPINK5 | 3 |
| BP | GO:0060740 | prostate gland epithelium morphogenesis | 0.002694 | 0.024337 | 0.017487 | SOX9/ID4 | 2 |
| BP | GO:0031345 | negative regulation of cell projection organization | 0.002697 | 0.024337 | 0.017487 | KLK8/LRP4/PTPRG/SEMA3E | 4 |
| BP | GO:0042475 | odontogenesis of dentin-containing tooth | 0.002759 | 0.024695 | 0.017745 | LRP4/TBX1/WDR72 | 3 |
| BP | GO:0022404 | molting cycle process | 0.002843 | 0.02505 | 0.017999 | SOX9/LRP4/SPINK5 | 3 |
| BP | GO:0022405 | hair cycle process | 0.002843 | 0.02505 | 0.017999 | SOX9/LRP4/SPINK5 | 3 |
| BP | GO:0061217 | regulation of mesonephros development | 0.002904 | 0.025394 | 0.018247 | SOX9/WNT2B | 2 |
| BP | GO:0098773 | skin epidermis development | 0.002928 | 0.025406 | 0.018256 | SOX9/LRP4/SPINK5 | 3 |
| BP | GO:0045185 | maintenance of protein location | 0.003015 | 0.025963 | 0.018655 | FBN2/IGSF11/MDFI | 3 |
| BP | GO:0060021 | roof of mouth development | 0.003104 | 0.026277 | 0.018881 | COL2A1/TBX1/TFAP2A | 3 |
| BP | GO:0060512 | prostate gland morphogenesis | 0.003122 | 0.026277 | 0.018881 | SOX9/ID4 | 2 |
| BP | GO:0097186 | amelogenesis | 0.003122 | 0.026277 | 0.018881 | TBX1/WDR72 | 2 |
| BP | GO:0060993 | kidney morphogenesis | 0.003194 | 0.026493 | 0.019036 | SOX9/IRX2/WNT2B | 3 |
| BP | GO:0072080 | nephron tubule development | 0.003194 | 0.026493 | 0.019036 | SOX9/IRX2/WNT2B | 3 |
| BP | GO:0048839 | inner ear development | 0.003284 | 0.02704 | 0.019429 | SOX9/COL2A1/TBX1/TFAP2A | 4 |
| BP | GO:0021602 | cranial nerve morphogenesis | 0.003346 | 0.027218 | 0.019557 | TBX1/TFAP2A | 2 |
| BP | GO:0001657 | ureteric bud development | 0.00338 | 0.027218 | 0.019557 | SOX9/EPCAM/WNT2B | 3 |
| BP | GO:0061326 | renal tubule development | 0.00338 | 0.027218 | 0.019557 | SOX9/IRX2/WNT2B | 3 |
| BP | GO:0070167 | regulation of biomineral tissue development | 0.003475 | 0.027218 | 0.019557 | SOX9/FBN2/TFAP2A | 3 |
| BP | GO:0072163 | mesonephric epithelium development | 0.003475 | 0.027218 | 0.019557 | SOX9/EPCAM/WNT2B | 3 |
| BP | GO:0072164 | mesonephric tubule development | 0.003475 | 0.027218 | 0.019557 | SOX9/EPCAM/WNT2B | 3 |
| BP | GO:0110149 | regulation of biomineralization | 0.003475 | 0.027218 | 0.019557 | SOX9/FBN2/TFAP2A | 3 |
| BP | GO:0050679 | positive regulation of epithelial cell proliferation | 0.003578 | 0.02746 | 0.019731 | SOX9/FZD7/TBX1/WNT2 | 4 |
| BP | GO:0061138 | morphogenesis of a branching epithelium | 0.003578 | 0.02746 | 0.019731 | SOX9/SEMA3E/WNT2/WNT2B | 4 |
| BP | GO:0003413 | chondrocyte differentiation involved in endochondral bone morphogenesis | 0.003579 | 0.02746 | 0.019731 | SOX9/VWA2 | 2 |
| BP | GO:0045844 | positive regulation of striated muscle tissue development | 0.00367 | 0.027782 | 0.019963 | ERBB3/TBX1/WNT2 | 3 |
| BP | GO:0048636 | positive regulation of muscle organ development | 0.00367 | 0.027782 | 0.019963 | ERBB3/TBX1/WNT2 | 3 |
| BP | GO:1901863 | positive regulation of muscle tissue development | 0.00377 | 0.028349 | 0.02037 | ERBB3/TBX1/WNT2 | 3 |
| BP | GO:0001823 | mesonephros development | 0.003871 | 0.028859 | 0.020736 | SOX9/EPCAM/WNT2B | 3 |
| BP | GO:0060541 | respiratory system development | 0.003889 | 0.028859 | 0.020736 | SOX9/CHI3L1/WNT2/WNT2B | 4 |
| BP | GO:0001708 | cell fate specification | 0.00408 | 0.030078 | 0.021613 | SOX9/FZD7/TBX1 | 3 |
| BP | GO:0060674 | placenta blood vessel development | 0.004319 | 0.031634 | 0.022731 | ITGB8/WNT2 | 2 |
| BP | GO:0001763 | morphogenesis of a branching structure | 0.004494 | 0.032659 | 0.023467 | SOX9/SEMA3E/WNT2/WNT2B | 4 |
| BP | GO:0055024 | regulation of cardiac muscle tissue development | 0.004517 | 0.032659 | 0.023467 | ERBB3/FZD7/WNT2 | 3 |
| BP | GO:0010092 | specification of animal organ identity | 0.00458 | 0.032906 | 0.023645 | WNT2/WNT2B | 2 |
| BP | GO:0030278 | regulation of ossification | 0.004637 | 0.033102 | 0.023786 | SOX9/FBN2/LRP4/TFAP2A | 4 |
| BP | GO:0060070 | canonical Wnt signaling pathway | 0.004705 | 0.033199 | 0.023855 | SOX9/FZD7/LRP4/WNT2/WNT2B | 5 |
| BP | GO:0071229 | cellular response to acid chemical | 0.004709 | 0.033199 | 0.023855 | SOX9/FZD7/TBX1/WNT2 | 4 |
| BP | GO:0048762 | mesenchymal cell differentiation | 0.004856 | 0.033855 | 0.024327 | SOX9/SEMA3E/TBX1/WNT2 | 4 |
| BP | GO:0072009 | nephron epithelium development | 0.004862 | 0.033855 | 0.024327 | SOX9/IRX2/WNT2B | 3 |
| BP | GO:0048048 | embryonic eye morphogenesis | 0.005124 | 0.035458 | 0.025479 | FBN2/TFAP2A | 2 |
| BP | GO:0060348 | bone development | 0.00516 | 0.03549 | 0.025502 | SOX9/COL2A1/TFAP2A/VWA2 | 4 |
| BP | GO:0003156 | regulation of animal organ formation | 0.005406 | 0.036737 | 0.026397 | WNT2/WNT2B | 2 |
| BP | GO:0060428 | lung epithelium development | 0.005406 | 0.036737 | 0.026397 | SOX9/WNT2 | 2 |
| BP | GO:0043583 | ear development | 0.005639 | 0.038088 | 0.027368 | SOX9/COL2A1/TBX1/TFAP2A | 4 |
| BP | GO:0030282 | bone mineralization | 0.00586 | 0.039348 | 0.028273 | SOX9/FBN2/TFAP2A | 3 |
| BP | GO:0009954 | proximal/distal pattern formation | 0.005992 | 0.039996 | 0.028739 | IRX2/LRP4 | 2 |
| BP | GO:0072089 | stem cell proliferation | 0.006264 | 0.041563 | 0.029865 | EPCAM/ID4/WNT2B | 3 |
| BP | GO:0048738 | cardiac muscle tissue development | 0.006502 | 0.042889 | 0.030818 | ERBB3/FZD7/NEBL/WNT2 | 4 |
| BP | GO:0090184 | positive regulation of kidney development | 0.006606 | 0.043323 | 0.03113 | SOX9/WNT2B | 2 |
| BP | GO:0014046 | dopamine secretion | 0.006923 | 0.044371 | 0.031883 | SYT1/SYT17 | 2 |
| BP | GO:0014059 | regulation of dopamine secretion | 0.006923 | 0.044371 | 0.031883 | SYT1/SYT17 | 2 |
| BP | GO:0030501 | positive regulation of bone mineralization | 0.006923 | 0.044371 | 0.031883 | FBN2/TFAP2A | 2 |
| BP | GO:0040019 | positive regulation of embryonic development | 0.006923 | 0.044371 | 0.031883 | WNT2/WNT2B | 2 |
| BP | GO:0030326 | embryonic limb morphogenesis | 0.007573 | 0.047752 | 0.034312 | FBN2/LRP4/TFAP2A | 3 |
| BP | GO:0035113 | embryonic appendage morphogenesis | 0.007573 | 0.047752 | 0.034312 | FBN2/LRP4/TFAP2A | 3 |
| BP | GO:0072210 | metanephric nephron development | 0.007578 | 0.047752 | 0.034312 | SOX9/IRX2 | 2 |
| BP | GO:0003007 | heart morphogenesis | 0.007749 | 0.048561 | 0.034893 | SOX9/COL2A1/TBX1/WNT2 | 4 |
| BP | GO:0014706 | striated muscle tissue development | 0.007899 | 0.048788 | 0.035057 | ERBB3/FZD7/NEBL/TBX1/WNT2 | 5 |
| BP | GO:0003197 | endocardial cushion development | 0.007915 | 0.048788 | 0.035057 | SOX9/ERBB3 | 2 |
| BP | GO:0048483 | autonomic nervous system development | 0.007915 | 0.048788 | 0.035057 | TBX1/TFAP2A | 2 |
| CC | GO:0005923 | bicellular tight junction | 3.82E-05 | 0.002353 | 0.00177 | AMOTL2/CLDN10/CLDN4/CLDN8/EPCAM | 5 |
| CC | GO:0070160 | tight junction | 4.56E-05 | 0.002353 | 0.00177 | AMOTL2/CLDN10/CLDN4/CLDN8/EPCAM | 5 |
| CC | GO:0016324 | apical plasma membrane | 6.56E-05 | 0.002353 | 0.00177 | AMOTL2/AQP6/CLDN4/EPCAM/ERBB3/PLD1/SLC34A2 | 7 |
| CC | GO:0043296 | apical junction complex | 7.47E-05 | 0.002353 | 0.00177 | AMOTL2/CLDN10/CLDN4/CLDN8/EPCAM | 5 |
| CC | GO:0045177 | apical part of cell | 0.000207 | 0.005213 | 0.00392 | AMOTL2/AQP6/CLDN4/EPCAM/ERBB3/PLD1/SLC34A2 | 7 |
| CC | GO:0062023 | collagen-containing extracellular matrix | 0.000249 | 0.005219 | 0.003924 | ADAMTS20/BCAM/COL2A1/FBN2/VWA2/WNT2/WNT2B | 7 |
| CC | GO:0060076 | excitatory synapse | 0.000447 | 0.00804 | 0.006045 | IGSF11/SLC6A9/SYT1 | 3 |
| CC | GO:0045178 | basal part of cell | 0.000616 | 0.009703 | 0.007296 | CLDN4/ERBB3/KRT14 | 3 |
| CC | GO:0016328 | lateral plasma membrane | 0.000714 | 0.009999 | 0.007518 | CLDN4/EPCAM/ERBB3 | 3 |
| CC | GO:0016327 | apicolateral plasma membrane | 0.001336 | 0.016835 | 0.012658 | CLDN4/CLDN8 | 2 |
| CC | GO:0005911 | cell-cell junction | 0.00177 | 0.020278 | 0.015247 | AMOTL2/CLDN10/CLDN4/CLDN8/EPCAM/IGSF11 | 6 |
| CC | GO:0098686 | hippocampal mossy fiber to CA3 synapse | 0.004264 | 0.040627 | 0.030547 | SLC6A9/SYT1 | 2 |
| CC | GO:0098984 | neuron to neuron synapse | 0.004321 | 0.040627 | 0.030547 | CRYAB/IGSF11/LRP4/SLC6A9/SYT1 | 5 |
| CC | GO:0030285 | integral component of synaptic vesicle membrane | 0.004514 | 0.040627 | 0.030547 | SLC6A9/SYT1 | 2 |
| CC | GO:0016323 | basolateral plasma membrane | 0.005183 | 0.043535 | 0.032733 | CLDN4/CLDN8/EPCAM/ERBB3 | 4 |
| CC | GO:0009925 | basal plasma membrane | 0.006153 | 0.048455 | 0.036432 | CLDN4/ERBB3 | 2 |
| MF | GO:0005544 | calcium-dependent phospholipid binding | 2.54E-05 | 0.003311 | 0.00274 | CPNE6/CPNE6/SYT1/SYT17 | 4 |
| MF | GO:0001786 | phosphatidylserine binding | 3.83E-05 | 0.003311 | 0.00274 | CPNE6/CPNE6/SYT1/SYT17 | 4 |
| MF | GO:0072341 | modified amino acid binding | 0.000191 | 0.011001 | 0.009103 | CPNE6/CPNE6/SYT1/SYT17 | 4 |
| MF | GO:0005109 | frizzled binding | 0.000343 | 0.01485 | 0.012288 | FZD7/WNT2/WNT2B | 3 |
| MF | GO:0001228 | DNA-binding transcription activator activity, RNA polymerase II-specific | 0.00055 | 0.016071 | 0.013299 | SOX9/EHF/ELF3/POU2F3/TBX1/TFAP2A/TLX1 | 7 |
| MF | GO:0001216 | DNA-binding transcription activator activity | 0.000557 | 0.016071 | 0.013299 | SOX9/EHF/ELF3/POU2F3/TBX1/TFAP2A/TLX1 | 7 |
