# Supplementary material for: Diagnostic and Prognostic Significances of SOX9 in Thymic Epithelial Tumor
Source: Front Oncol. 2021 Oct 28;11:708735. doi: 10.3389/fonc.2021.708735 (PMC8580949; doi:10.3389/fonc.2021.708735)
Supplement: Supplementary file 8 [file Table_5.docx]

Supplementary Table 7. JASPAR analysis results for SOX9 binding sites located within the promoter of the *POU2F3* gene (Sequence ID: NC_000011.10 :120234638-120232638)

| Relative score | Start | End | Strand | Predicted sequence |
| --- | --- | --- | --- | --- |
| 0.870 | 1549 | 1557 | - | CTTTTGTTT |
| 0.857 | 846 | 854 | - | TCATTGTCC |
| 0.844 | 814 | 822 | - | TCATTGATA |
| 0.815 | 408 | 416 | - | CCATTGGGT |
| 0.800 | 1813 | 1821 | + | ATATAGTTC |
